# Perspective: Why and How Ubiquitously Distributed, Vascular-Associated, Pluripotent Stem Cells in the Adult Body (vaPS Cells) Are the Next Generation of Medicine

**DOI:** 10.3390/cells10092303

**Published:** 2021-09-03

**Authors:** Eckhard U. Alt, Christoph Schmitz, Xiaowen Bai

**Affiliations:** 1 Heart and Vascular Institute, Department of Medicine, Tulane University School of Medicine, New Orleans, LA 70112, USA; xibai@mcw.edu; 2 Sanford School of Medicine, University of South Dakota, Sioux Falls, SD 57104, USA; 3 MD Anderson Cancer Center, University of Texas, Houston, TX 77054, USA; 4 Isar Klinikum Munich, 80331 Munich, Germany; 5 Chair of Neuroanatomy, Institute of Anatomy, Faculty of Medicine, Ludwig-Maximilians University of Munich, 80336 Munich, Germany; christoph_schmitz@med.uni-muenchen.de; 6 Department of Cell Biology, Neurobiology & Anatomy, Medical College of Wisconsin, Milwaukee, WI 53226, USA

**Keywords:** adipose-derived regenerative cells, adipose-derived stem cells, bone regeneration, cartilage regeneration, regenerative medicine, scar tissue, tendon regeneration, wound healing

## Abstract

A certain cell type can be isolated from different organs in the adult body that can differentiate into ectoderm, mesoderm, and endoderm, providing significant support for the existence of a certain type of small, vascular-associated, pluripotent stem cell ubiquitously distributed in all organs in the adult body (vaPS cells). These vaPS cells fundamentally differ from embryonic stem cells and induced pluripotent stem cells in that the latter possess the necessary genetic guidance that makes them intrinsically pluripotent. In contrast, vaPS cells do not have this intrinsic genetic guidance, but are able to differentiate into somatic cells of all three lineages under guidance of the microenvironment they are located in, independent from the original tissue or organ where they had resided. These vaPS cells are of high relevance for clinical application because they are contained in unmodified, autologous, adipose-derived regenerative cells (UA-ADRCs). The latter can be obtained from and re-applied to the same patient at the point of care, without the need for further processing, manipulation, and culturing. These findings as well as various clinical examples presented in this paper demonstrate the potential of UA-ADRCs for enabling an entirely new generation of medicine for the benefit of patients and healthcare systems.

## 1. Introduction

Regenerative cell therapy, which refers to the therapeutic application of stem cells to repair diseased or injured tissue, has received increasing attention from basic scientists, clinicians, and the public. Stem cells hold significant promise for tissue regeneration due to their innate ability to provide a renewable supply of cells that can form multiple cell types, whole tissue structures, and even organs. Stem cells are present in the human body at all stages of life from the earliest times of an embryo through adulthood and senescence.

The aim of this paper is to summarize results of landmark experiments on stem cells and stem cell therapy performed in the labs of the authors over the last two decades (ethical approvals are summarized in the “Institutional Review Board Statement” section at the end of this paper), and to discuss the results of this research in the context of the literature dedicated to stem cells and stem cell therapy. Considering the fact that a search in PubMed on “stem cells” on 6 August 2021 yielded over 385,000 citations (among them more than 63,000 reviews), and a search on “stem cell therapy” approximately 140,000 citations (among them more than 35,500 reviews), it becomes obvious that this paper provides a perspective rather than a systematic review.

### 1.1. The Current State of Confusion about the Term Stem Cells in the Literature

Approximately 50 years ago, Friedenstein et al. [1,2] described cells derived from bone marrow stroma that were adherent to plastic and had the ability to differentiate into cells other than hematopoietic lineages. Since then, our knowledge about stem cells has been steadily growing.

Nature portfolio [3] has provided the following definition of the term pluripotent stem cells: “Pluripotent stem cells are cells that have the capacity to self-renew by dividing and to develop into the three primary germ cell layers of the early embryo and therefore into all cells of the adult body, but not extra-embryonic tissues such as the placenta. Embryonic stem cells and induced pluripotent stem cells are pluripotent stem cells” [3]. In line with this, the California Institute for Regenerative Medicine (CIRM) has provided a similar definition of the term pluripotent stem cells [4]: “Pluripotent means “many potentials”. In other words, these cells have the potential of taking on many forms in the body, including all of the more than 200 different cell types. Embryonic stem cells are pluripotent, as are induced pluripotent stem (iPS) cells that are reprogrammed from adult tissues. When scientists talk about pluripotent stem cells, they mostly mean either embryonic or iPS cells” [4]. A very similar definition was also provided by the International Society for Stem Cell Research, ISSCR [5]). Several studies demonstrated the generation of viable mice by tetraploid complementation, in which mouse iPS cells were injected into a tetraploid blastocyst to allow them to fully express their developmental potential [6,7].

Of note, this definition of the term pluripotent stem cells excludes the presence of any form of pluripotent stem cell in the adult body, implying that there are no cells in the adult body that could differentiate into all cells of the adult body.

In line with this, the CIRM has provided the following definition of the term adult stem cells [4]: “Adult stem cells are found in the various tissues and organs of the human body. They are thought to exist in most tissues and organs where they are the source of new cells throughout the life of the organism, replacing cells lost to natural turnover or to damage or disease. Adult stem cells are committed to becoming a cell from their tissue of origin, and can’t form other cell types. They are therefore also called tissue-specific stem cells. They have the broad ability to become many of the cell types present in the organ they reside in” [4]. A very similar definition was also provided by ISSCR [5]. This definition contrasts with the definition of the term multipotent mesenchymal stromal cells (MSCs), as provided in a position statement by The International Society for Cellular Therapy (ICST) in 2006 [8] as “being adherent to plastic, expressing the surface markers CD73, CD90 and CD105, and having the ability to differentiate into osteoblasts, adipocytes and chondrocytes” [8]. In fact, we and many others have shown that cells can be isolated from bone marrow and vessel walls of adults that have the capacity to differentiate (upon stimulation, but without genetic reprogramming) into many more cell types than osteoblasts, adipocytes, and chondrocytes [9,10,11]. Consequently, a recent study defined microvascular pericytes with the ability to produce somatic cells representative for the three primitive germ layers (ectoderm, mesoderm, and endoderm) as pluripotent adult stem cells [11], which is contrasts with the definitions provided by Nature portfolio [3] and the CIRM [4]. In fact, the situation is even much more complicated considering that several other surface markers of MSCs next to CD73, CD90, and CD105 were described, including (in alphabetical order) CD49f, CD146, CD200, CD271, CD349, GD2, MSCA-1, PODXL, Sox11, SSEA-3, SSEA-4, Stro-1, Stro-4, SUSD2, TM4SF1, and 3G5 (summarized in [12]). This list was established based on reports of cells isolated from many different tissues, including (in alphabetical order) adipose tissue, amnion, bone marrow, decidua parietalis, dental pulp, dermis, endometrium, periodontal ligament, placenta, umbilical cord, and umbilical cord blood. However, not each of these surface markers was identified on MSCs isolated from each of the tissues listed above. In addition, some of these markers were only found after cultivating cells for up to 100 days [12].

### 1.2. Introduction of the Term Vascular-Associated, Pluripotent Stem Cells in the Adult Body (vaPS Cells)

We will use the following terminology throughout this paper. (i) A certain cell type can be isolated from different organs in the adult body (i.e., adipose tissue, heart, skin, bone marrow, or skeletal muscle) that can differentiate into ectoderm, mesoderm, and endoderm, providing significant support for the existence of a certain type of small, ubiquitously distributed, universal, vascular-associated, pluripotent stem cell in the adult body (vaPS cells). (ii) These vaPS cells fundamentally differ from embryonic stem cells and iPS cells in that the latter possess the necessary genetic guidance that makes them intrinsically pluripotent. In contrast, vaPS cells do not have this intrinsic genetic guidance. Nevertheless, they are able to differentiate into somatic cells of all three lineages under guidance of the microenvironment they are located in, independent from the original tissue or organ that they are derived from. (iii) As vaPS cells are contained in adipose-derived regenerative cells (ADRCs), the latter are able to form any somatic cell lineage guided by the respective tissue or organ they are applied to without the need for prior genetic modification. (iv) A cellular preparation that results from culturing fresh, unmodified ADRCs is called adipose-derived stem cells (ADSCs).

Note that some authors argued that pericytes would be the ancestors of perivascular MSCs [11,13,14], which would be in contrast to the concept of vaPS cells, as outlined in this paper. However, pericytes are fully differentiated cells that already have a terminal, differentiated purpose in life, namely the formation of capillaries together with endothelial cells [15]. Two recent findings challenge the concept that pericytes would be the ancestors of perivascular MSCs: (i) culturing human ADSCs in a specific pericyte medium can induce pericyte-like differentiation of the ADSCs [16]; and (ii) neuron-glial antigen 2 (NG2), which has long been associated with pericytes [13,14,17], was recently identified as a consistent surface marker of long-living human cord blood mesenchymal stem cells (LL-cbMSCs) that were fully characterized according to ISCT [8], and, to a lesser degree, of human bone marrow mesenchymal stem cells [18] (vaPS cells were not investigated in this study). NG2 was also identified in extracellular vesicles produced by LL-cbMSCs [18]. These data support the hypothesis that at least a subset of vaPS cells is also immunopositive for NG2 and, thus, NG2 is expressed by more cells than just by pericytes.

The reason why both vaPS cells and pericytes express NG2 may be explained by the fact that both cells must be in close contact to the (abluminal side of the) endothelial basal lamina. Specifically, vaPS cells are able travel to their destination via adjacent tissue and the blood stream upon activation [19,20], and the roles of pericytes in forming the typical capillary structure together with endothelial cells [15,21,22] and vessel regulation [23] require that they are located close to the endothelial basal lamina. This may be achieved by expression of NG2, as NG2 binds to Type VI collagen through the central nonglobular domain of its core protein [24], and Type VI collagen anchors endothelial basement membranes by interacting with Type IV collagen [25].

### 1.3. Need for Introducing the Term vaPS Cells

As defined above, vaPS cells are neither pluripotent stem cells as defined by CIRM [4] nor MSCs, as defined by ICST [8] and ISSCR [5]. Specifically, vaPS cells do not yet express CD73, CD90, and CD105, as outlined in detail in the following section.

Of note, this paper does not describe a new cell type that has not been investigated before. Rather, in our opinion, vaPS cells were investigated in many studies in the past but were somewhat incorrectly attributed to MSCs.

Furthermore, it should be mentioned that other authors described very small cells with reduced metabolic activity and pluripotent potential in the adult body in the past [26,27]. However, these cells were not described in the literature as ubiquitously distributed and vascular-associated.

## 2. The Ubiquitous Occurrence of Stem Cells in Tissue That Contains Blood Vessels

Blood vessels are the initial structures to be formed when a new organ is developing in an embryo. The presence of vaPS cells in the vascular location allows equal distribution of stem cells with pluripotent capacity (except for forming placental tissue) throughout the body. These cells are assumed to serve as a repertoire for renewal of the respective tissue and organs for the rest of the life of the individual.

For example, a certain number of stem cells per gram tissue can be isolated from rat brain tissue. However, when preparing a microvascular preparation of rat brain tissue—in which only microvessels were remaining and the rest of the brain tissue was discarded—we found that the resulting number of stem cells per gram tissue increased by several potencies, indicating that the majority of stem cells are indeed located or associated with the vascular structure (Alt, Bai et al.; unpublished results). Moreover, we were able to demonstrate that the same vaPS cells can be isolated from blood vessels independent of the organ or tissue they are derived from (outlined in detail below).

### 2.1. Existence of Mesenchymal Stem Cells in Blood Vessels

**Figure 1:**
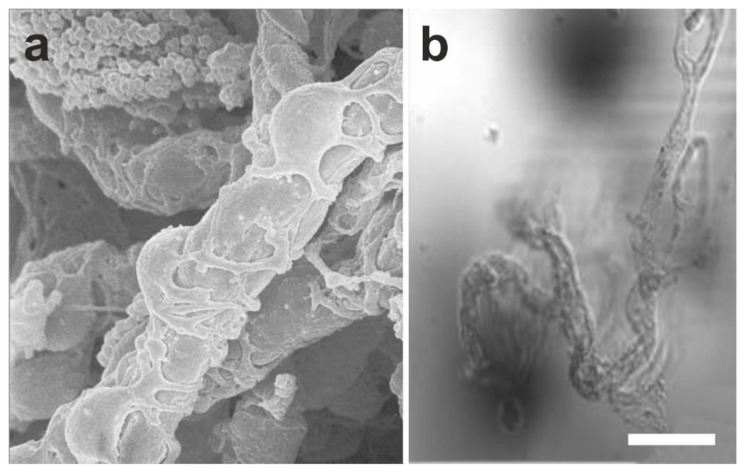
Microvessel-associated cells (microvessel isolated from a rat brain) represent a universal resource within the entire body (previously unpublished figure). (**a**) Scanning electron microscopic image of a microvessel. (**b**) Phase contrast image of a microvessel as used for further analysis. The scale bar represents 5 µm in (**a**) and 40 µm in (**b**).

Results of studies from our laboratories demonstrate that vaPS cells can be isolated from all blood vessels, independent of the organ or tissue that they are derived from. This was demonstrated with cells derived from both human and animal tissue. Specifically, these vaPS cells were isolated from microvessels. Figure 1 shows a microvessel preparation and a phase contrast image of such a microvessel from a rat brain.

**Figure 2:**
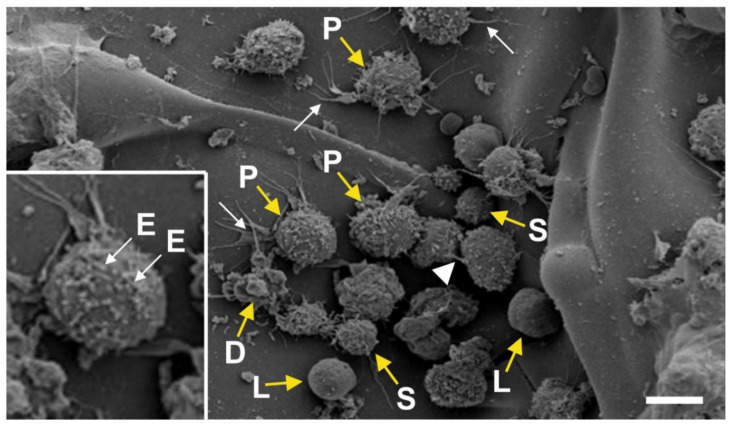
Scanning electron microscopic image of cells that were freshly isolated from human abdominal adipose tissue (reprinted from [31] with permission from the sportärztezeitung). Abbreviations: P, progenitor cells; S, small cells; D, dying cells; L, lymphocytes; E, exosomes. The white arrows point to actin filaments, and the white arrowhead to a micro-channel between two cells. Details are provided in the main text. The scale bar represents 10 µm (20 µm in the high-power inset).

Figure 2 shows a scanning electron microscopic image of cells that were freshly isolated from human abdominal adipose tissue by enzymatic release, and were imaged just minutes after isolation and plating. These cells (which represent the ADRCs) were not cultured but freshly isolated. The presence of a composition of different cells is found in this image. One can recognize larger cells that exhibit a rough surface structure (‘P’ in Figure 2). These cells are progenitor cells that have already started to build actin filaments (white arrows in Figure 2) in order to enhance their adherence to the extracellular matrix on which they were seeded. Besides this, some of these cells have started to communicate through microtubular structures (arrowhead in Figure 2).

It should be mentioned that, in contrast to stem cells, progenitor cells are already on a pre-determined pathway to become a differentiated cell and have lost their ability to decide what they want to be ‘in life’. Accordingly, progenitor cells are typically determined to differentiate and develop into a lineage defined cell type. For example, in bone marrow, more than 99% of the cells are not stem cells, but primarily hematopoietic progenitor cells [28,29]. Accordingly, progenitor cells have already started a pathway of lineage-committed differentiation. In the case of bone marrow-derived cells, these hematopoietic progenitor cells (often incorrectly labeled as stem cells) started to differentiate into future hematopoietic cells of the white, red, or platelet lineage. Pending on their progress in maturation in this differentiation process, these cells are no longer able to significantly revert their pathway of differentiation. At best, they are able to vary somewhat within the same germ layer of differentiation, but typically stay within the same lineage [30].

In addition, there are also lymphocytes present in the cellular preparation that was freshly isolated from human abdominal adipose tissue by enzymatic release (‘L’ in Figure 2), as well as small cells (‘S’ in Figure 2). These small cells show a relatively smooth surface and are limited yet in their ability to adhere to the extracellular matrix. They do not show exosomal structures on their surface as some of the larger cells do (‘E’ in the high-power inset in Figure 2). In addition, dying cells are found which are devolving into apoptotic bodies (‘D’ in Figure 2).

Analyzing the individual cells shown in Figure 2 in more detail revealed that approximately 20% of the cells were ‘naked’ and smooth on their surface, most likely representing white blood cells such as lymphocytes (‘L’ in Figure 2). The content of the small assumed vaPS cells (‘S’ in Figure 2) was about 10% of the cell population shown in Figure 2. This demonstrates that, following enzymatic preparation, ADRCs of human abdominal adipose tissue are composed of vaPS cells, white blood cells, and larger cells, most likely progenitor cells. However, the composition between the different cellular elements varies with the tissue these cells are obtained from.

**Figure 3:**
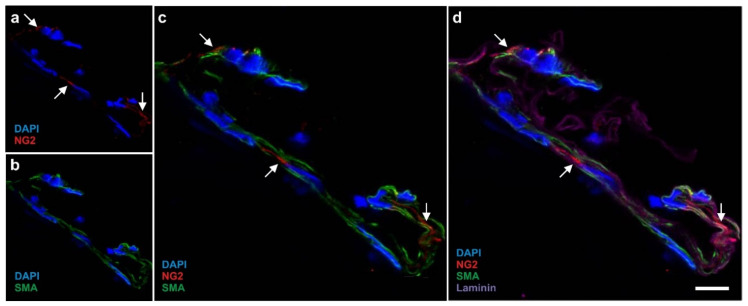
Immunofluorescence detection of neuron-glial antigen 2 (NG2) (red in (**a**,**c**,**d**); arrows), smooth muscle antigen (SMA) (green in (**b**–**d**) and laminin (purple in (**d**) in the wall of a small human arteriole (cell nuclei in blue) (previously unpublished figure). The scale bar represents 20 µm in (**a**,**b**) and 10 µm in (**c**,**d**).

A more precise location of the cells in the vascular structures is revealed by multi-colored immunohistochemistry of a small arteriole (Figure 3). Cell nuclei (blue in Figure 3) and smooth muscle antigen (SMA) (green in Figure 3) define the structure of the arteriole. Furthermore, the location of laminin (purple in Figure 3) correlates with the border between the endothelial basal lamina (representing the intima) and the media containing the smooth muscle cells (note that in this position also the internal elastic lamina is found [32]). Of note, NG2 (red in Figure 3) was found in close proximity to laminin.

**Figure 4:**
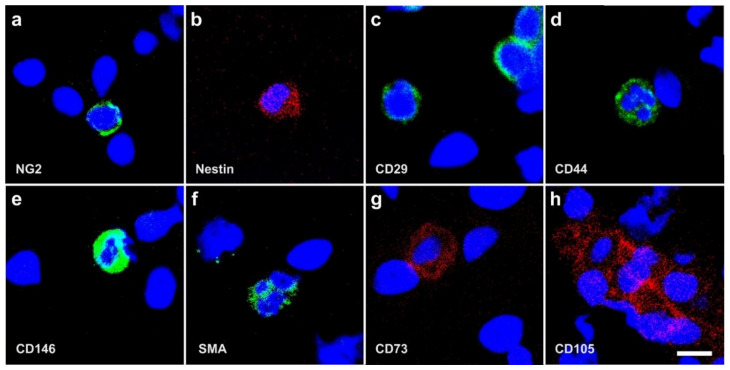
Immunofluorescence detection of NG2 (**a**), Nestin (**b**), CD29 (**c**), CD44 (**d**), CD146 (**e**), smooth muscle antigen (SMA) (**f**), CD73 (**g**), and CD105 (**h**) in cells that were freshly isolated from human adipose tissue (previously unpublished figure). Note the very small cytosol of the cells that were immunopositive for NG2, Nestin, and CD29 (**a**–**c**). The scale bar represents 10 µm in (**a**–**h**).

Using immunofluorescence we were able to detect NG2, Nestin, CD29, CD44, CD146, smooth muscle antigen (SMA), CD73, and CD105 in human cells that were freshly isolated from adipose tissue (Figure 4). Of note, cells immunopositive for NG2, Nestin, and CD29 showed a very small cytosol (Figure 4a–c).

Nestin is an early marker of neural stem/progenitor cells as well as of proliferative endothelial cells [33]. These cells have a tiny cytosol compared to the nucleus and to other, more differentiated cells. Furthermore, cells immunopositive for CD29 (integrin β1) exhibit this kind of small cytosolic immunostaining (Figure 4c). Together with CD49e (integrin α-5) CD29 forms integrin α5β1, the primary receptor for fibronectin [34,35,36]. Most probably, the latter attaches the vaPS cells to the extracellular matrix within the vessel wall. This is supported by the fact that SPARC (secreted protein acidic and rich in cysteine; also known as osteonectin) [37,38] can mobilize ADSCs through its effect on integrin α5β1 [39,40], providing a functional basis for the regulation of the contribution of these cells to tissue and organ repair by SPARC. The latter is synthesized by several types of cells, including osteoblasts and odontoblasts [41], as well as endothelial cells and fibroblasts [42], but also macrophages [43], infiltrating leukocytes [44] and cancer cells [45]. Thus, SPARC may represent a key regulator in making vaPS cells a replacement source responsive to the signals of the surrounding tissue.

It is of note that SPARC is also expressed by ADSCs in vitro [46]. Moreover, the SPARC-related modular calcium-binding protein 1 (SMOC1), a member of the SPARC family and serving as a regulator of osteoblast differentiation, was found in the secretome of bone marrow-derived MSCs [47]. Thus, SPARC may play a pivotal role in both affecting the properties of vaPS cells in terms of proliferation and differentiation based on cues from the extracellular environment, as well as in paracrine activities of vaPS cells, impacting upon the activities of other cells in the local microenvironment [48]. 

Other stainings and flow cytometric analyses showed that vaPS cells are additionally positive for Oct4 [49], Sca1 [50], and SSEA4 [51]).

### 2.2. The Hypothesis of a Universal, Vascular-Associated Stem Cell

**Figure 5:**
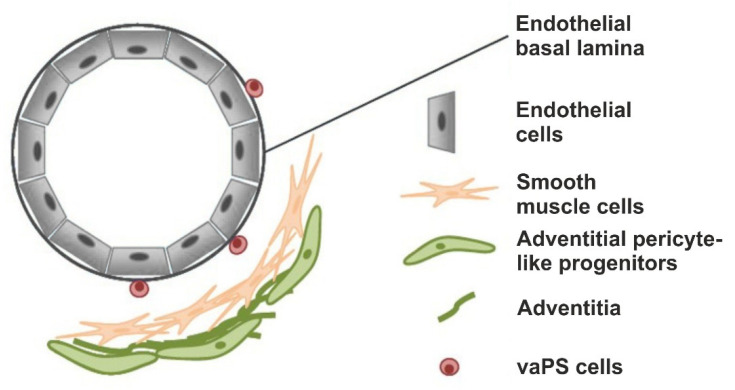
Schematic illustration of the hypothesized location of the vaPS cells (adapted from [54,55] with permission from John Wiley & Sons—Books and MDPI).

**Figure 6:**
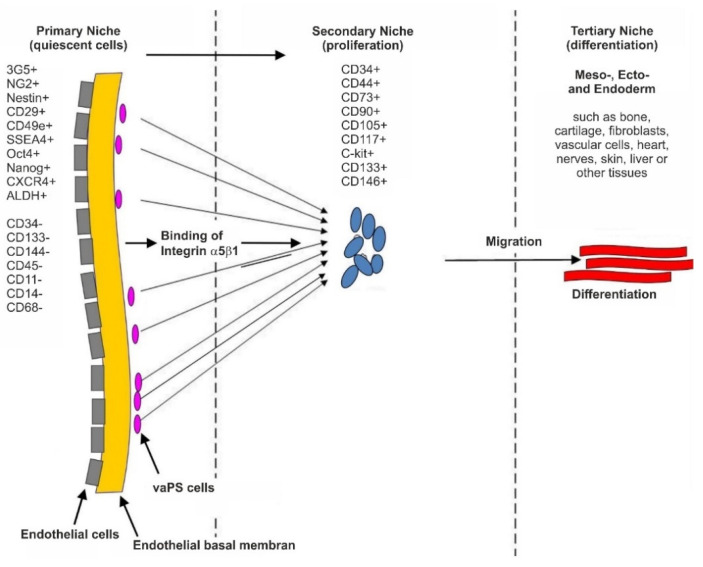
Expression of surface markers of vaPS cells in their primary, secondary and tertiary niche (previously unpublished figure).

Figure 5 and Figure 6 illustrate our hypothesis regarding the location and surface marker expression of vaPS cells. Figure 5 shows the endothelial basal lamina in black and the endothelial cells on the luminal side of the endothelial basal lamina in blue. We hypothesize that the vaPS cells reside opposite to the endothelial cells on the abluminal side of the endothelial basal lamina, towards the smooth muscle cell layer and embedded within those. We are aware of the fact that this is in contrast to other descriptions in the literature that vascular-associated MSCs, presumably immunopositive for pericyte markers, would be located in the adventitia [14,52,53] (addressed in detail above).

The vaPS cells are small, which allows them to migrate through tissue in order to help maintaining tissue homeostasis [54,55]. When these cells leave their quiescent location (i.e., their primary niche, as depicted in Figure 6), they start to migrate, followed by proliferation which is primarily under control of the Wnt signaling pathway [56,57,58], and finally differentiation. The location of proliferation may be called the secondary niche (Figure 6), in contrast to the primary niche that is assigned to the vessel wall (Figure 5 and Figure 6). In this regard, we hypothesize that markers such as CD44, CD73, CD90, and CD105—often believed to be indicative of stem cells [59]—are only present in cells that have already left their primary niche and started to enter the next developmental phase in order to attain progenitor status. This hypothesis is supported by the fact that CD44, CD90, and CD105 are also expressed by fibroblasts that exhibit no plasticity at all [49]. Finally, the cells can leave their secondary niche and differentiate in the tertiary niche (Figure 6) into their final lineage. The tissue specific differentiation pathway is controlled by signaling from the cells’ new microenvironment, including micro-RNA and transcription factors [60,61,62]. Accordingly, throughout life, replacing cells exist that can be mobilized upon need for tissue renewal and repair.

### 2.3. Surface Markers of vaPS Cells Cultured in Serum-Free Media

**Figure 7:**
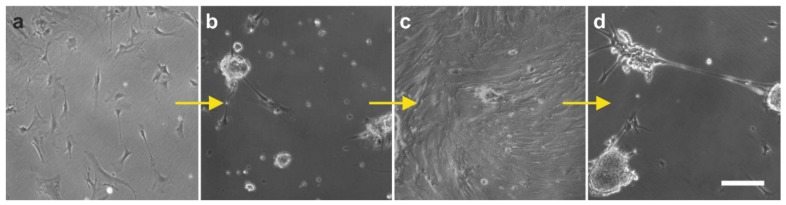
Change in the morphology of rat adipose-derived stem cells cultured first in fetal bovine serum (FBS) for 14 days (**a**), then in serum-free medium (SFM) for 24 h (**b**), and then again in FBS for one week (**c**). Afterwards, transfer to SFM again induced a spheroid-like appearance (**d**) (previously unpublished figure). The arrows indicate the consecutive order of the panels. The scale bar represents 100 µm.

**Figure 8:**
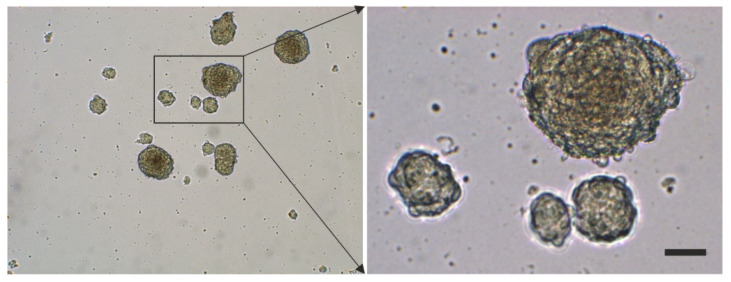
Aggregation of human adipose-derived stem cells, leading to the formation of spheroids that resemble the appearance of embryoid bodies formed by embryonic stem cells (reprinted from [10] with permission from PLoS). The scale bar represents 50 µm in the right panel.

When cultured in fetal bovine serum (FBS), ADSCs display a typical, spindle-shaped appearance (Figure 7a,c). In contrast, they form spheroids when cultured in serum-free media [SFM] (Figure 7b,d). After culturing ADSCs for seven days in SFM, we found spheroid bodies with a diameter of up to 250 µm [10] (Figure 8). The shape of these spheroid bodies resembled pretty much the shape of spheroid bodies formed by embryonic stem cells, known as embryoid bodies [63]. 

**Figure 9:**
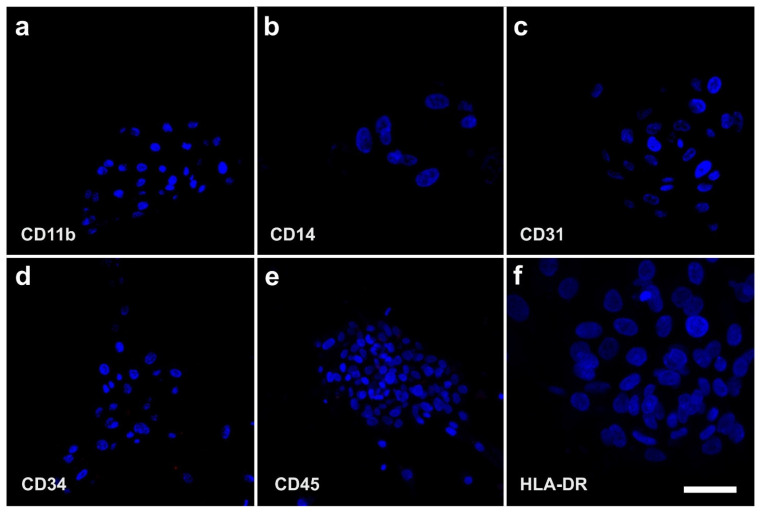
Immunofluorescence detection of CD11b (**a**), CD14 (**b**), CD31 (**c**), CD34 (**d**), CD45 (**e**), and HLA-DR (**f**) on the surface of human adipose-derived stem cells that were cultured for four days in serum-free media (previously unpublished figure). The scale bar represents 60 µm in (**a**,**d**,**e**), 20 µm in (**b**), 40 µm in (**c**), and 30 µm in (**f**).

Immunofluorescence analysis of human ADSCs that were cultured for four days in SFM showed that these cells were immunonegative for CD11b (a marker of macrophages), CD14 (a marker of hematopoietic progenitor cells), CD31 (a marker of endothelial progenitor cells), CD34 (a marker of progenitor cells in general), CD45 (a pan-leukocyte marker), and HLA-DR (Figure 9). Corresponding flow cytometric analysis revealed that the relative numbers of cells that were immunopositive for these surface markers were smaller than 1.5% (relative number of cells that were immunopositive for CD11b (CD11b+): 1.1%; CD14+: 0.6%; CD31+: <0.1%; CD34+: 0.4%; CD45+: 1.3% and HLA-DR: 0.2%) [9,49,64]. These results confirmed that, after culturing ADSCs for four days in SFM, macrophages, hematopoietic progenitor cells, endothelial progenitor cells, progenitor cells in general, cells expressing the pan-leukocyte marker CD45, and cells expressing HLA-DR were virtually absent in this cell culture. Therefore, culturing ADSCs in SFM is a powerful tool to discriminate between vaPS cells, progenitor cells, and differentiated cells. We hypothesize that the loss of CD34+ cells after culturing ADSCs for four days in SFM is a consequence of missing signaling from the normally surrounding microenvironment in the natural tissue.

**Figure 10:**
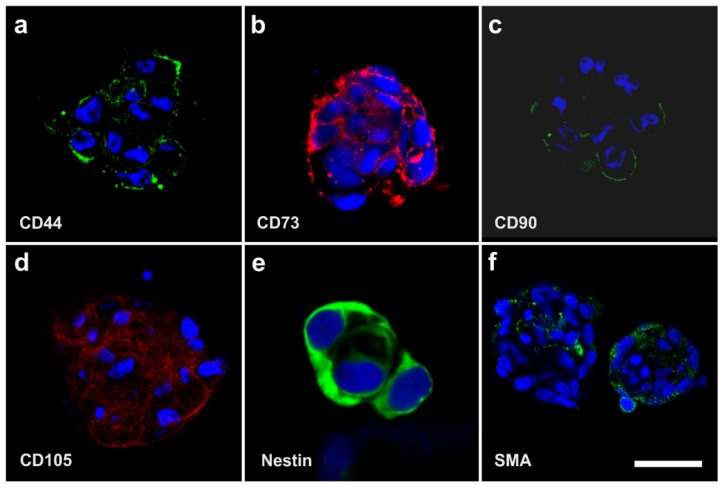
Immunofluorescence detection of CD44 (**a**), CD73 (**b**), CD90 (**c**), CD105 (**d**), Nestin (**e**), and smooth muscle antigen (SMA) (**f**) on the surface of human adipose-derived stem cells that were cultured for four days in serum-free media (previously unpublished figure). The scale bar represents 16 µm in (**a**,**c**), 12 µm in (**b**,**d**,**f**), and 20 µm in (**e**).

In contrast, Figure 10 shows the positive surface marker antigen profile of human ADSCs that were cultured for four days in SFM. Immunofluorescence analysis showed that these cells were immunopositive for CD44, CD73, CD90, CD105, Nestin, and SMA. Corresponding flow cytometric analysis revealed the following relative numbers of immunopositive cells in this culture: CD44 (CD44+): 92.4%, CD73+: 99.1%, CD90+: 28.8%, CD105+: 73.3%, Nestin+: 76.2%, and SMA+: 41.2%. After 120 h in culture, the relative number of CD105+ cells increased to 96.7% [9,49,64]. These data are in line with results reported by other authors [65]. 

**Figure 11:**
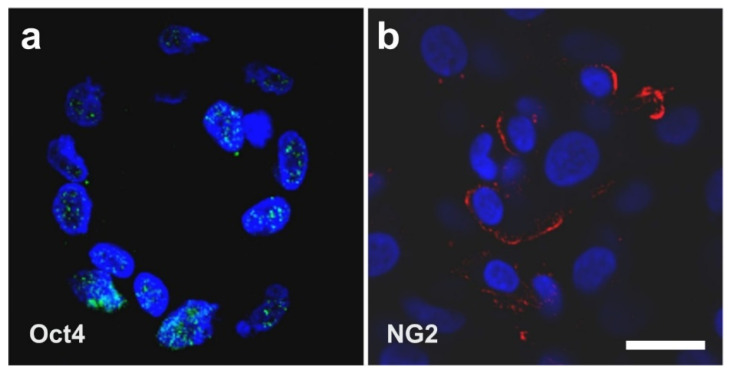
Immunofluorescence detection of Oct4 (**a**) and neuron-glial antigen 2 (NG2) (**b**) in cells in spheroids that were created by unmodified human adipose-derived stem cells that were cultured for four days in serum-free media (previously unpublished figure). The scale bar represents 20 µm.

Most importantly, in spheroids that were created from unmodified human ADSCs that were cultured for four days in SFM, cells expressed Oct4 as an indicator of ‘stemness’ as well as NG2 (Figure 11) (note that no colocalization analysis was performed). This indicates the natural presence of (vascular associated) stem cells in the body without the need for prior genetic modification of the cells as in case of iPS cells (as explained in detail above the results shown in Figure 11 cannot be explained by contamination with circulating cells that express CD34 and CD45). 

It should be noted that the expression of Oct4 and NG2 in stem cells isolated from vessel walls was also reported by other authors [66]; in the latter study, these cells were isolated from human post-mortem arterial segments that were stored in a tissue-banking facility for at least five years [66].

### 2.4. The Pluripotency of vaPS Cells

It has been questioned whether adult pluripotent stem cells (as defined above) exist, or if the differentiation into the three germ layers is based on the presence of a composition of different progenitor cells that are responsible for the individual differentiation capacity into the respective lineage. To answer this question, we performed two key experiments.

**Figure 12:**
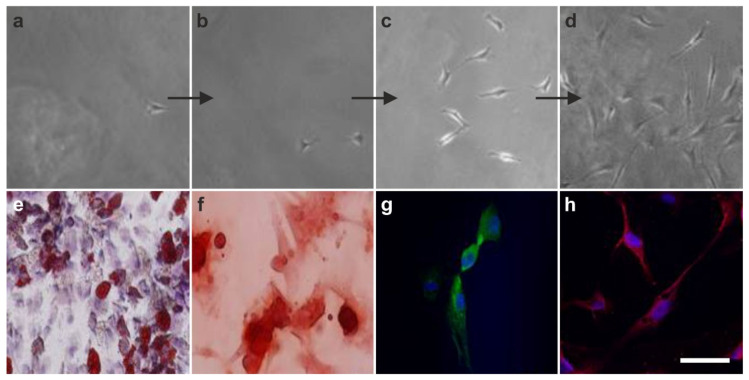
Clonal expansion of a single human vaPS cell (i.e., a single human ADSC) for five days in fetal bovine serum (FBS), followed by multilineage differentiation induced by culturing the cells for three weeks in the respective induction media (protocols are provided in [10]).(previously unpublished figure). (**a**–**d**) Culturing of cells in FBS for 6 h (**a**), 20 h (**b**), 48 h (**c**), and five days (**d**). (**e**–**h**) Induction of adipogenesis (mesoderm) (**e**), osteogenesis (mesoderm) (**f**), hepatogenesis (endoderm) (**g**), and neurogenesis (ectoderm) (**h**), confirmed by oil red O staining (**e**) and alizarin red S staining (**f**) as well as immunofluorescence detection of albumin (specific for hepatocytes) (**g**) and microtubule-associated protein 2 (MAP-2) (specific for neurons) (**h**). The scale bar represents 80 µm in (**a**–**d**), 100 µm in (**e**,**f**), and 40 µm in (**g**,**h**).

In the first key experiment, a single human vaPS cell (i.e., a single human ADSC) was clonally expanded for five days in FBS, resulting in proliferation at a doubling time of about 24 h into millions of cells (Figure 12a–d). Then, cells were separated from this clonally expanded culture and subjected to adipogenic, osteogenic, hepatogenic, and neurogenic induction media. It was found that the clonally expanded cells, which all expressed the same genotypic profile, were able to differentiate into ectoderm, mesoderm, and endoderm (Figure 12e–h), indicating that the initial single cell was indeed an adult stem cell with pluripotent potential.

**Figure 13:**
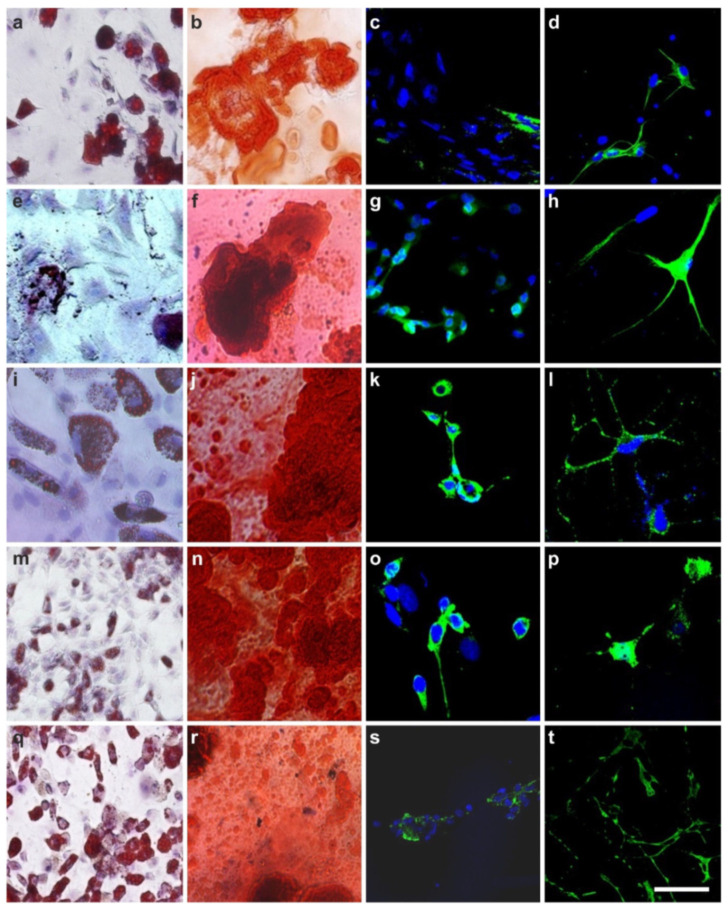
Induction of adipogenesis (mesoderm; confirmed by oil red O staining) (**a**,**e**,**i**,**m**,**q**), osteogenesis (mesoderm; confirmed by alizarin red S staining) (**b**,**f**,**j**,**n**,**r**), hepatogenesis (endoderm; confirmed by immunofluorescent detection of albumin) (**c**,**g**,**k**,**o**,**s**), and neurogenesis (ectoderm; confirmed by immunofluorescent detection of MAP-2) (**d**,**h**,**l**,**p**,**t**) by culturing rat vaPS cells obtained from adipose tissue (**a**–**d**), heart (**e**–**h**), skin (**i**–**l**), bone marrow (**m**–**p**), and skeletal muscle (**q**–**t**) for three weeks in the respective induction media (previously unpublished figure). The scale bar represents 100 µm in (**a**,**e**,**m**,**q**), 120 µm in (**b**), 50 µm in (**c**,**d**,**f**–**h**,**j**,**n**,**r**), 20 µm in (**i**), 70 µm in (**k**), 30 µm in (**l**,**p**), 25 µm in (**o**), and 60 µm in (**s**,**t**).

In the second key experiment, we isolated vaPS cells from different organs (adipose tissue, heart, skin, bone marrow, and skeletal muscle) of rats and subjected them after proliferation in FBS to adipogenic, osteogenic, hepatogenic, and neurogenic induction media. Again, the cells were able to differentiate into ectoderm, mesoderm, and endoderm (Figure 13).

The results of these key experiments provide significant support for the hypothesis of a universal, vascular-associated stem cell with pluripotent potential.

### 2.5. The Three Germ Layer Differentiation Potential of Human Adipose-Derived Stem Cells

**Figure 14:**
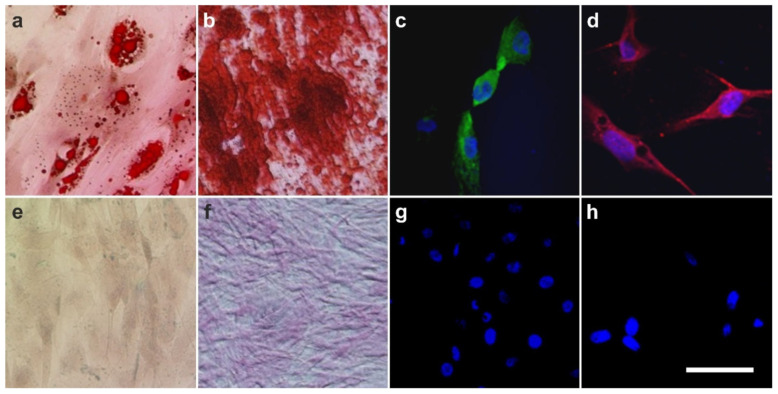
Induction of adipogenesis (mesoderm; confirmed by oil red O staining) (**a**), osteogenesis (mesoderm; confirmed by alizarin red S staining) (**b**), hepatogenesis (endoderm; confirmed by immunofluorescent detection of albumin) (**c**) and neurogenesis (ectoderm; confirmed by immunofluorescent detection of MAP-2) (**d**) by culturing human adipose-derived stem cells (ADSCs) for three weeks in the respective induction media (previously unpublished figure). No induction of three germ layer differentiation was observed by culturing human ADSCs in non-inductive control media (**e**–**h**). The scale bar represents 100 µm in (**a**,**b**,**e**,**f**) and 40 µm in (**c**,**d**,**g**,**h**).

In 2007, we initially demonstrated the three germ layer differentiation potential of human ADSCs into adipocytes, osteoblasts, hepatocytes, and neurons [67]. While the cells cultured in non-inductive media did not attain the lineage specific expression, cells subjected to the specific induction media demonstrated the respective differentiation (Figure 14).

### 2.6. Integration of vaPS Cells into Host Tissue upon Activation

**Figure 15:**
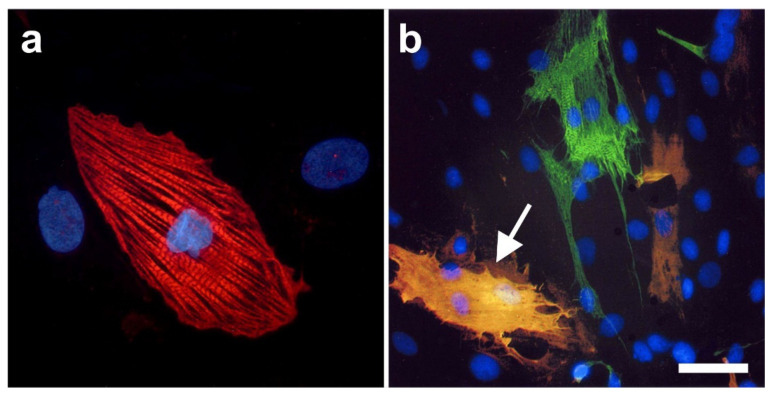
(**a**) Immunofluorescence detection of troponin T in the cytoplasm of a rat cardiomyocyte (red signal) (adapted from [68] with permission from John Wiley & Sons—Books) Human adipose-derived stem cells that were labeled with green fluorescent protein (GFP) (green signal) (previously unpublished figure). The arrow points to a GFP-positive cell that additionally was immunopositive for MF20 (resulting in a yellowish signal). The scale bar represents 20 µm in (**a**) and 40 µm in (**b**).

The first experiments we conducted in this regard were carried out to highlight the possible influence of the microenvironment surrounding the vaPS cells. This involved co-culturing of neonatal rat cardiomyocytes with human ADSCs together with fusion-inducing hemagglutinating virus of Japan (HVJ). In order to discriminate between the two different types of cells, we labeled the human ADSCs with green fluorescent protein (GFP). Figure 15a shows a cell that is immunopositive for the cardiac-specific protein, troponin T (red signal). Figure 15b shows GFP (green signal) in the cytosol of human ADSCs (after treatment with HVJ and, after five days of culture time, we observed a fusion efficiency of approximately 20% [68]). One cell shows a yellowish cell body (arrow) that was obtained by the overlay of the green signal (GFP) with a red signal originating from immunofluorescence detection of MF20, one of the early markers of the cardio-myogenic pathway differentiation [69]. In contrast, ADSCs that were not co-cultured with rat cardiomyocytes did not show this early cardiomyogenic pathway differentiation.

**Figure 16:**
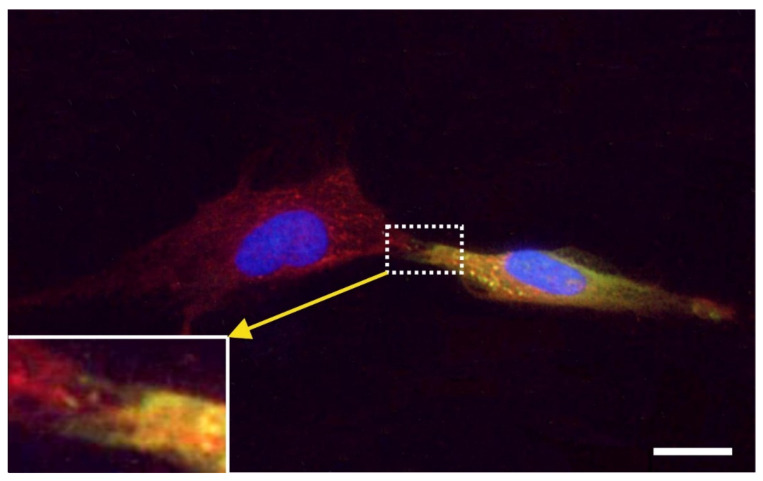
Cell–cell contact between a rat cardiomyocyte (expressing troponin T; red signal) and a cell expressing both green fluorescent protein and troponin T (yellowish signal), representing a human adipose-derived stem cell at the latest stage of culturing (adapted from [68] with permission from John Wiley & Sons—Books). The inset shows the contact between the cells at higher magnification. The scale bar represents 15 µm (5 µm in the inset).

Figure 16 shows a direct cell–cell contact between a rat cardiomyocyte (identified by immunofluorescence detection of troponin T; red signal) and a cell that expressed both GFP and troponin T (yellowish signal). The latter represents a human ADSC at the latest stage of culturing. At higher magnification (inset in Figure 16), it appears that the two cells adhered to each other. 

We also demonstrated that five days after treatment with fusion-inducing HVJ, human ADSCs that were fused with rat cardiomyocytes showed spontaneous rhythmic contraction and exhibited action potential [68].

**Figure 17:**
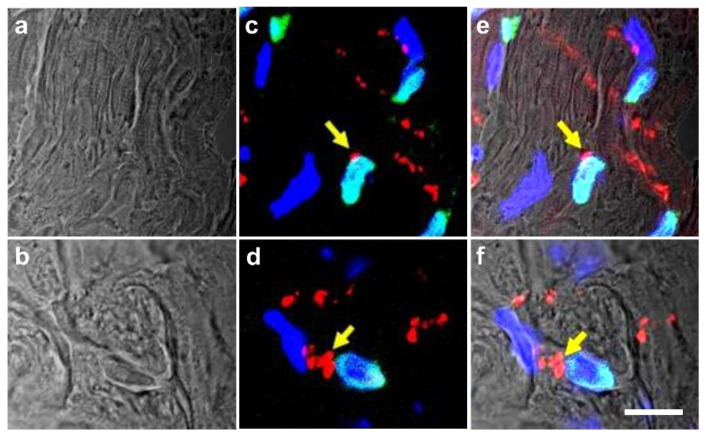
Phase-contrast images (**a**,**b**), fluorescence images (**c**,**d**) and merged images (**e**,**f**) of heart sections prepared four weeks after experimental induction of myocardial infarction in severe combined immunodeficient (SCID) mice and injection of human adipose-derived stem cells (ADSCs) (**a**,**c**,**e**) or human adipose-derived regenerative cells (ADRCs) (**b**,**d**,**f**) into the peri-infarct region (adapted from [9] with permission from Oxford University Press). Cell nuclei were labeled with DAPI (blue); nuclei of human cells were labeled with an antibody against human lamin A/C (green); and gap junctions were labeled with an antibody against connexin 43 (red). Yellow arrows indicate detection of connexin 43 in gap junctions that could not be attributed to cell–cell contacts between mouse cells but most probably represented cell–cell contacts between mouse cardiomyocytes and descendants of injected human cells. The scale bar represents 15 µm in (**a**) and 10 µm in (**b**).

In order to demonstrate that both ADSCs and ADRCs integrate into host tissue after transplantation in vivo and form adequate contacts with cells of the host tissue, we experimentally induced myocardial infarction in severe combined immunodeficient (SCID) mice and injected human ADRCs or human ADSCs into the peri-infarct region [9]. Four weeks later, the myocardial function was improved (evidenced by improved mean ejection fraction (*p* < 0.01) and reduced mean end-systolic volume (*p* < 0.01) compared to injection of saline). At that time, grafted ADRCs and ADSCs had undergone cardiomyogenic differentiation, as indicated by expression of connexin 43 and troponin I in a fusion-independent manner (Figure 17).

**Figure 18:**
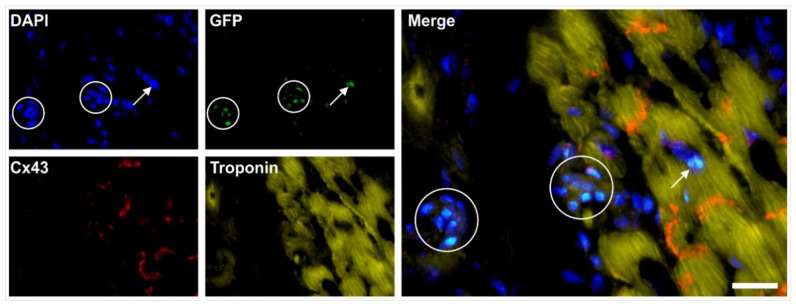
Representative photomicrographs of a paraffin-embedded, 5-µm-thick tissue section of a post-mortem heart from a pig, taken from the left ventricular border zone of myocardial infarction ten weeks after experimental occlusion of the left anterior descending (LAD) artery for three hours, followed by the delivery of eGFP-labeled autologous adipose-derived stem cells into the balloon-blocked LAD vein (matching the initial LAD occlusion site) at four weeks after occlusion of the LAD (previously unpublished figure). The section was stained with DAPI (blue) and processed for immunofluorescence detection of GFP (green), connexin 43 (Cx43) (red), and troponin (yellow). The circles indicate regions where most of the cell nuclei were immunopositive for GFP, and the arrow a GFP-positive cell nucleus inside a cardiomyocyte. The scale bar represents 25 µm in the merged panel, and 50 µm in the individual panels.

Finally, in order to demonstrate that ADSCs or their descendants differentiate into functional cells of the host tissue in vivo, we induced myocardial infarction in pigs by experimental occlusion of the left anterior descending (LAD) artery for three hours, followed by the delivery of eGFP-labeled autologous ADSCs into the balloon-blocked LAD vein (matching the initial LAD occlusion site) at four weeks after occlusion of the LAD [70]. Six weeks later, the animals were sacrificed and sections of the heart were stained with DAPI and processed for immunofluorescence detection of GFP, connexin 43, and troponin. Cell nuclei immunopositive for GFP were found in the wall of small vessels as well as in cardiomyocytes (Figure 18). 

Pigs that were treated identically except for injection of ADRCs (which cannot be labeled by definition) instead of ADSCs showed statistically significant improvements in cardiac function and structure as well, compared to the injection of saline [70].

### 2.7. Exchange of Information between vaPS Cells and Other Cells in Cell Culture

**Figure 19:**
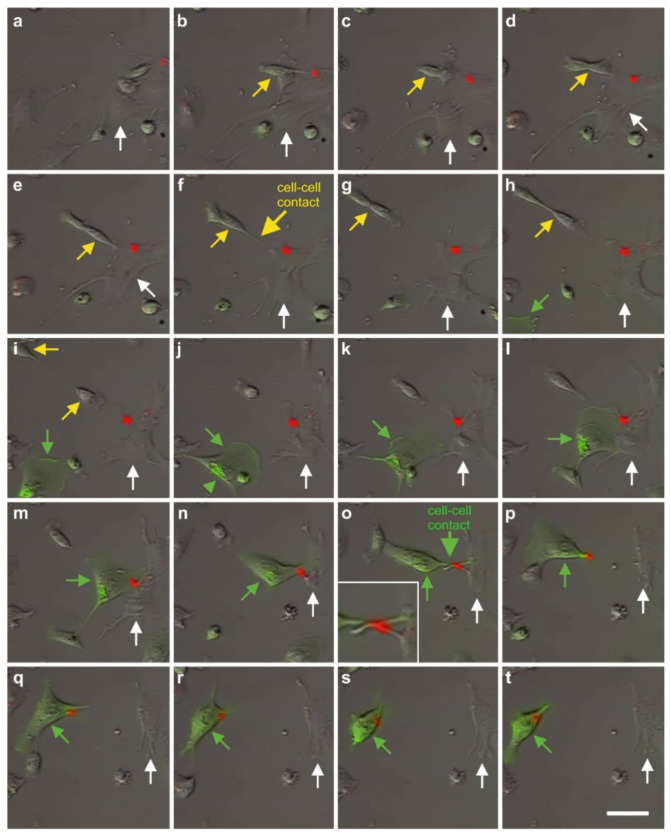
Time-lapse video microscopy of human adipose-derived stem cells (ADSCs) that were co-cultured with MDA-MB-231 cells labeled with green fluorescent protein (GFP) (green signal in (**h**–**t**)) (previously unpublished figure). One of the ADSCs shown in this figure was labeled with a red quantum dot (red signal in all panels; the corresponding cell body is marked by a white arrow in all panels). In (**f**), this ADSC formed a contact with another ADSC (yellow arrows in (**b**–**i**); the cell–cell contact is indicated in (**f**). However, the quantum dot was not exchanged between the cells. In (**o**) the same ADSC formed a contact with a MDA-MB-231 cell (green arrows in (**h**–**t**)); the cell–cell contact is indicated in (**o**) and the quantum dot was transferred from the ADSC to the MDA-MB-231 cell. The high-power inset in (**o**) shows that the quantum dot left the ADSC through a microchannel that was formed by the ADSC itself, which demonstrates active participation of the ADSC in exchange of cellular components between the cells. The time interval between the frames was 40 min each. The scale bar represents 50 µm in (**a**–**t**), and 25 in the inset in (**o**).

We also investigated the exchange of information between vaPS cells (i.e., ADSCs) and other cells in cell culture by time-lapse video microscopy of human ADSCs that were labeled with red quantum dots [71] and were co-cultured with MDA-MB-231 cells (a commercially available human breast cancer cell line; [72]) that were labeled with GFP (Figure 19). In our opinion, the communication mechanism between cells found in this experiment is the same as the cell–cell connection of endothelial progenitor cells with cardiac myocytes by nanotubes described in [73] as an important mechanism for cell fate changes.

**Figure 20:**
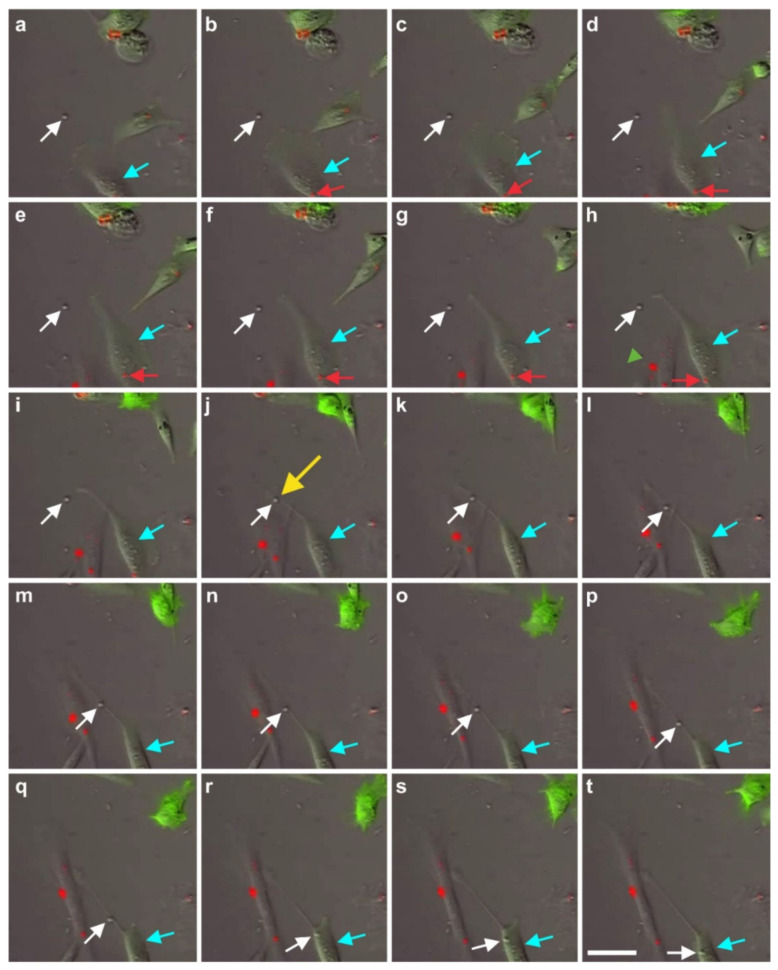
Time-lapse video microscopy of human adipose-derived stem cells (ADSCs) that were co-cultured with MDA-MB-231 cells labeled with green fluorescent protein (GFP) (green signal in all panels) (previously unpublished figure). Several of the ADSCs shown in this figure were labeled with red quantum dots (red signal in all panels). A quantum dot in the cell body of one of the ADSCs is marked by a red arrow in (**b**–**h**); the corresponding cell is marked by a blue arrow in all panels. The white arrow in all panels point to an exosome. The yellow arrow in (**j**) indicates the event of pinocytosis of this exosome by the ADSC that is marked by the blue arrow. The time interval between the frames was 20 min each. The scale bar in (**t**) represents 50 µm.

A second type of cell–cell communication is based on genetic information contained in exosomes (or microsomes), which are released from the cell surface (c.f. the inset in Figure 2) and, upon uptake by pinocytosis (Figure 20), induce a certain epigenetic reprogramming in the recipient cell [74,75,76]. These exosomes are currently of major interest with respect to further elucidating cell–cell communication [77]. Specifically, communication through the content of exosomes is considered an important factor for orientation of cells [78]. Several micro-RNAs, transcription factors, and cytokines were identified to be involved in the transfer of epigenetic reprogramming of cells in order to direct them into a specific lineage differentiation, further supporting the hypothesis that the differentiation pathway of vaPS cells upon activation is controlled by signaling from the cells’ new microenvironment [60,61,62].

### 2.8. Immunosuppressive and Anti-Inflammatory Activities of ADSCs

**Figure 21:**
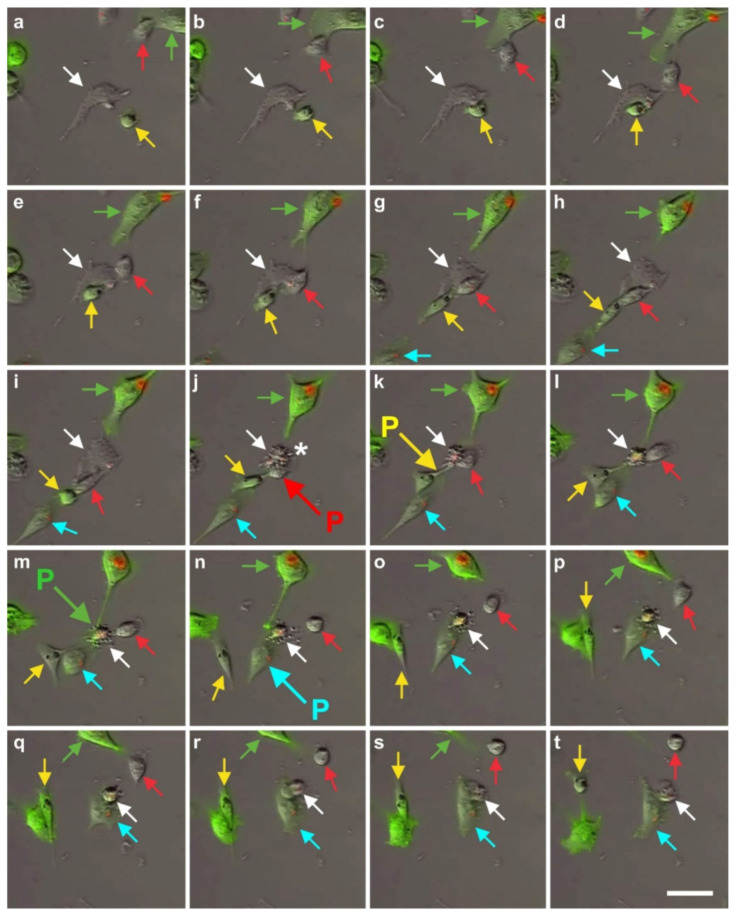
Time-lapse video microscopy of human adipose-derived stem cells (ADSCs) that were co-cultured with MDA-MB-231 cells labeled with green fluorescent protein (GFP) (green signal in all panels; the red signal in the panels came from red quantum dots that were exchanged between the ADSCs and the MDA-MB-231 cells (c.f. Figure 19) (previously unpublished figure). The white arrow in all panels indicates an ADSC that underwent apoptosis (named “white cell”; the disintegration of the cell body of the white cell is marked in (**j**) by an asterisk). The red, yellow, green and blue arrows indicate four other cells (named “red cell”, “yellow cell”, “green cell”, and “blue cell”) that migrated towards the white cell during the three hours before disintegration of the cell body of the white cell (**a**–**i**). The red cell was an ADSC whereas the yellow, green and blue cells were MDA-MB-231 cells. During the first 90 min after disintegration of the cell body of the white cell the other cells took up apoptotic bodies of the white cell by means of pinocytosis (indicated by a “P” in the respective color in (**j**,**k**,**m**,**n**)). The easiest way to recognize these pinocytosis events is to sequentially follow each cell through the panels (**a**–**t**). The time interval between the frames was 20 min each. The scale bar in (**t**) represents 50 µm.

ADSCs exhibit potent immunosuppressive and anti-inflammatory activities [79,80], and exosomes were shown to play an important role in these processes [81]. In recent years, apoptotic bodies, a major class of extracellular vesicles released as a product of apoptotic cell disassembly, have become recognized as another key player in immune modulation [81]. A recent study demonstrated that apoptosis in human bone marrow-derived MSCs induced recipient-mediated immunomodulation in vivo [82]. On the other hand, even in tissues with high cellular turnover, apoptotic cells are rarely seen because of efficient clearance mechanisms, including the sensing of cells that undergo apoptosis via ‘find me’ signals (i.e., chemotactic factors) [83]. One of these chemotactic factors is the phospholipid known as lysophosphatidylcholine [84]. Increased concentration of lysophosphatidylcholine was found in the medium in which hematopoietic progenitor cells underwent apoptosis following growth factor withdrawal [85]. Our time-lapse video microscopic investigations showed that human ADSCs that undergo apoptosis also stimulate the migration of other cells to the apoptotic cell, and these other cells can then take up the apoptotic bodies via pinocytosis (Figure 21). However, it is currently unknown which ‘find me’ signals are used by ADSCs that undergo apoptosis. In any case, apoptosis of ADSCs may be a key event in immunosuppressive and anti-inflammatory activities mediated by these cells.

## 3. A Key Role of Stem Cell Therapy in Re-Establishing Tissue Homeostasis between Dying and Replacing Cells

**Figure 22:**
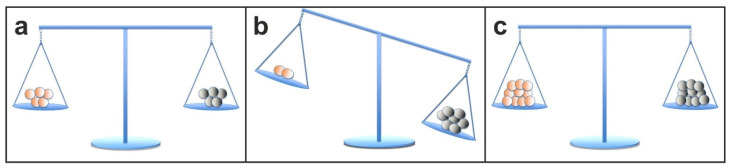
Homeostasis between dying cells (gray) and stem cells (orange) in tissue under healthy conditions (**a**), the situation under pathological conditions as well as during aging (**b**), and after stem cell therapy (**c**) (previously unpublished figure). Under healthy conditions, tissue homeostasis is maintained between dying cells and replacing stem cells. In contrast, tissue homeostasis is disrupted under pathological conditions as well as during aging as more cells are dying than being replaced by stem cells. Application of a concentrated stem cell preparation can re-establish tissue homeostasis.

A key function of stem cells in the adult body is to contribute to the homeostasis of tissue resident parenchymal cells. As we age, there is a continuous turnover in almost every tissue between dying and replacing cells (with the exception of some nerve cells in the brain, which will not be discussed in detail here; c.f. [86,87,88]). For a long time our body can maintain tissue homeostasis; the equilibrium between dying cells and stem cells is depicted in Figure 22a. However, tissue homeostasis can be disturbed with increasing age in all tissues, such as tendons, bone, joints, heart, liver, kidneys, and muscles, in a way that the *parenchymal* cells, which are responsible for the organ function, are more frequently replaced by *mesenchymal fibroblastic* cells. This is due to a lack of renewing power, especially if ischemia, infections, accidents, and other inflammatory or traumatic events accelerate the tissue turnover (Figure 22b). A good example are chronic wounds that show a number of problems, including insufficient levels of cell proliferation, increased cell senescence/apoptosis, impaired angiogenesis/neovascularization, inflammation, increased production of matrix metalloproteinases (MMPs), increased matrix degradation, and decreased production of extracellular matrix [88]. 

An important question that has remained in this regard is the following: why do injured organs (for example, a heart after a myocardial infarction) not source the stem cells from a part of the body where they are present and would not be ‘missed’ after recruitment (such as from adipose tissue)? It is currently difficult to provide a satisfactory answer to this question, because it would require to analyze model organisms in which spontaneous recruitment of stem cells from other sites of the body (where they are present in larger numbers and would not be ‘missed’ after recruitment) would occur. To our knowledge, such model organisms are currently less studied. Nevertheless, it might be postulated that the release and activation of dormant stem cells from their local position within vessel walls by the osteonectin signaling (as proteins are cleaved relatively fast by proteases) might be primarily confined to the immediate vicinity of the initial stem cell location.

Several animal models for the study of limb regeneration have been described, among them the Mexican axolotl (Ambystoma mexicanum) [89,90,91,92]. It turned out that. in limb regeneration, a morphologically uniform intermediate (the so-called blastema) is formed, consisting of a variety of stem and progenitor cells originating from a variety of tissues [93]. Further deciphering the genetic and molecular regeneration inducers that are involved in limb regeneration [94,95] may serve as the basis to understand which signals could, in general, be used by injured organs to recover stem cells from parts of the body where they are not ‘missed’ after recruitment, followed by investigations into why this does not happen in the human body. In any case, the distance between the stem cells in these parts of the body where they would not be ‘missed’ after recruitment (as in adipose tissue) and those cells that ‘call’ for the stem cells by the release of cytokines may simply be too long. 

Stem cell therapy is to be considered as the principal of transferring concentrated stem cells, which have been taken from one part of the body where they are not ‘missed’, to tissue in need of regeneration, in order to re-establish tissue homeostasis (Figure 22c). The isolation of stem cells from suitable tissue (such as adipose tissue) and their application to other injured tissue and organs can be interpreted as the most gentle and natural approach to help the body in self-repair by increasing the number of stem cells at a location where they are exhausted, but most needed. From these considerations, it also becomes clear that stem cell therapy is not only directed to a specific organ, tissue or disease, but will take the function of replacing and repairing tissue and organs that suffer from a lack of repair, renewal, and rejuvenation.

## 4. The Significance of vaPS Cells and iPS Cells for the Practice of Medicine

The results presented above challenge the somewhat incorrect public belief that naturally no cells would exist in the adult body that are able to differentiate into all three lineages without being first (genetically) modified. The latter may have substantially contributed to the euphoria around iPS cells (in which first an artificial (induced) overexpression of embryonic genes, such as Oct4, Klf4, Sox2, and/or cMyc is necessary [96,97,98]), which resulted in granting Dr. Shin’ya Yamanaka (University of Kyoto, Japan) the Nobel Prize in Medicine 2012. One key motivation of this paper was to summarize evidence demonstrating that there are indeed cells in the adult body that are able to differentiate into all three lineages (i.e., the vaPS cells), and, at this time, there is no evidence that vaPS cells could not develop into all cells of the adult body. Hence, iPS cells may not be required for the general practice of medicine. 

Furthermore, there are concerns that iPS cells can demonstrate features similar to cancer cells [99,100,101]. Dr. Paul Knoepfler and his team at UC Davis School of Medicine (Davis, CA, USA) were the first to demonstrate that induced pluripotency and oncogenic transformation are related processes when comparing the transcriptomes of iPS cells with the transcriptomes of cancer cells [102,103,104]. Hence, the iPS cells technology will most likely not advance to a stage where therapeutic transplants are necessarily deemed safe [105]. On the other hand, iPS cells are supportive in helping to better understand differentiation pathways of stem cells and patient-specific bases of diseases, as well as to develop personalized drug discovery efforts [106].

In light of these considerations, the clinical significance of vaPS cells is outlined in detail in the following sections.

## 5. Key Advantages of Adipose-Derived Regenerative Cells and Adipose-Derived Stem Cells for Cell Based Therapies

### 5.1. Comparison of Bone Marrow Derived Stem Cells with Adipose-Derived Regenerative Cells and Adipose-Derived Stem Cells

For almost a decade bone marrow was the primary source of stem cells for research into and development of therapies based on stem cells isolated from the adult body. Bone marrow-derived stem cells exhibit significant potential for promoting tissue regeneration, protection of ischemic tissue at risk, and modulation of inflammation and autoimmunity [107]. However, utilizing bone marrow-derived stem cells for therapeutic purposes typically requires to first isolate these cells and expand them in culture. Because of the overwhelming presence of hematopoietic progenitor cells in bone marrow that aim to form new blood cells [108], only a small fraction of the cells in fresh bone marrow aspirate are stem cells. In contrast, other tissues, such as adipose tissue, yield orders of magnitude higher numbers of stem cells per unit volume than bone marrow [29,109]. Thus, ADRCs may be utilized as a fresh cell preparation, rich in vaPS cells, without the need for expansion in cell culture. 

Compared to other sources of stem cells in the adult body, adipose tissue has the following specific advantages [55]: (i) adipose tissue is readily available in most individuals; (ii) small amounts of adipose tissue (25 to 100 mL) can be harvested using a mini-liposuction procedure with low invasiveness, with tolerable discomfort and low donor-site damage; (iii) considerably larger amounts of stem cells can be obtained from adipose tissue than from the same amount of bone marrow; and (iv) ADRCs can be used in clinical applications without further need of culturing (as in case of ADSCs). Given these advantages, unmodified, autologous ADRCs (UA-ADRCs) appear to be the most promising candidate for repair and regeneration of many tissues, including chronic wounds, soft tissue defects, bone and cartilage defects, non-healing fractures, injured tendons, diseased or injured myocardium, urological conditions such as incontinence, and neurological conditions [55,110,111,112,113,114].

### 5.2. Differences in the Effectiveness of Various Systems and Methods That Are Available for Isolating Adipose-Derived Regenerative Cells 

Different techniques and protocols were described for releasing ADRCs for therapeutic use [10,115,116,117]. Collagenase I and II containing enzyme preparations that degrade collagen are commonly used. However, in order to release the vaPS cells from their binding site in the extracellular matrix inside the blood vessels (and hereby to release the cells from their ‘hibernating’ or silenced state), collagenases are only partially effective. The addition of a neutral protease to a collagenase enzyme preparation can significantly increase the number of ADRCs recovered from a given volume of adipose tissue [10]. This was achieved by developing the proprietary Matrase^®^ enzymatic reagent (InGeneron Inc., Houston, TX, USA) [10]. Isolating ADRCs with the Matrase enzymatic reagent and the Transpose RT^®^ system (InGeneron) appears advantageous to other commercial cell separation systems [10]. Specifically, ADRCs that were isolated with the Matrase enzymatic reagent and the Transpose RT system may contain approximately 40% of cells that are immunopositive for CD29 and CD44, which are markers of ADSCs [70]. The latter authors also reported a colony-forming units frequency (CFU-F) (considered to be an indicator of stemness) of approximately 11% of ADRCs isolated with the Matrase enzymatic reagent and the Transpose RT system. Other authors reported relative CFU-F values of approximately 8% when isolating ADRCs from equine adipose tissue using the same technology [118]. In contrast, relative CFU-F values between 0.2% and 1.7% were reported for ADRCs that were isolated in head-to-head comparisons with four other commercial cell separation systems that do not make use of neutral protease [119,120]. However, a direct comparison of the CFU-F values is hardly possible due to significant methodological differences surrounding how the respective CFU-F values were determined.

### 5.3. Long-Term Survival of Adipose-Derived Stem Cells after Transplantation in Animal Models into the Heart and Subcutaneous Locations

**Figure 23:**
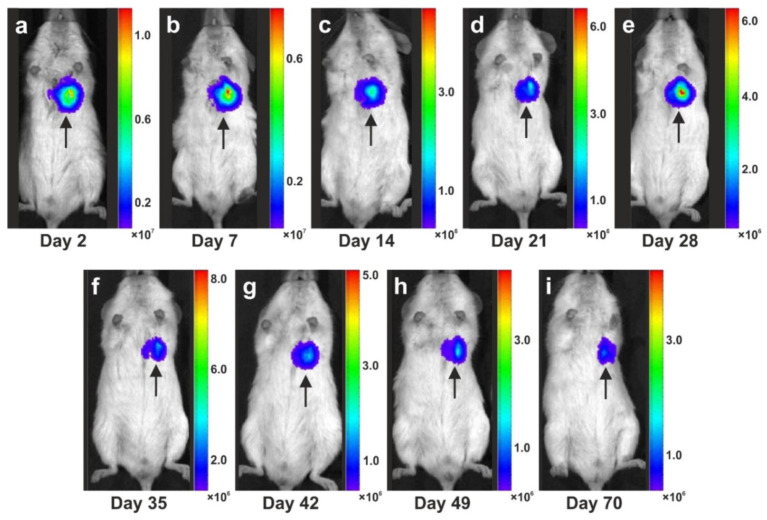
In vivo bioluminescence imaging over time of 5 × 10^5^ human adipose-derived stem cells (ADSCs) that were intramyocardially delivered into SCID mice after experimental induction of myocardial infarction by permanent ligation of the left anterior descending coronary artery (adapted from [123] with permission from Springer Nature). The ADSCs were transfected with a lentiviral vector expressing green fluorescent protein and luciferase. Strong bioluminescence signals were found over the heart region at all investigated time points (arrows), indicating the long-term survival of delivered ADSCs in injured hearts. The investigated time points are provided below the panels.

**Figure 24:**
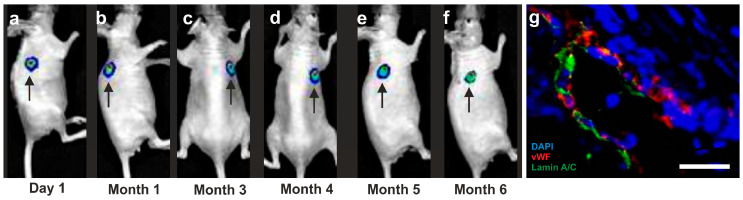
(**a**–**f**) In vivo bioluminescence imaging over time of human adipose-derived stem cells (ADSCs) that were delivered together with crosslinked collagen into a subcutaneous location of SCID mice (previously unpublished figure). The ADSCs were transfected with a lentiviral vector expressing green fluorescent protein and luciferase. Strong bioluminescence signals were found at the injection site up to 6 month (arrows), indicating the long-term survival of delivered ADSCs in subcutaneous tissue. (**g**) Immunofluorescence detection of von Willebrand factor (vWF; red signal) and Lamin A/C (green signal) as an indicator of human cells in a SCID mouse, forming a blood vessel four weeks after application of the human ADSCs into a subcutaneous wound (counterstaining with DAPI; blue signal). The scale bar represents 20 µm in (**g**).

In order to study the capacity of ADSCs to survive for a long time at the site of engraftment, we transfected human ADSCs with a lentiviral vector expressing GFP and luciferase (when the luciferase enzyme is expressed in living cells, these cells are capable of converting systemically injected luciferin dye into an active luminescent fluorophor that can be detected noninvasively [121,122]). Figure 23 and Figure 24 show the time course of luciferin positive human ADSCs that were either intramyocardially delivered into SCID mice after experimental induction of myocardial infarction by permanent ligation of the left anterior descending coronary artery (Figure 23), or into a subcutaneous location of SCID mice (Figure 24), respectively. Strong bioluminescence signals were found over the injection sites at all investigated time points (up to 70 days post-delivery in Figure 23 and up to six months post-delivery in Figure 24; further time points were not investigated), demonstrating the long-term survival of ADSCs delivered into injured hearts or at subcutaneous locations, respectively. At no other location of the body of the SCID mice, a cell engraftment was detected.

To better understand the fate of the delivered ADSCs, we investigated subcutaneous tissue harvested at the injection site at four weeks after subcutaneous application of human ADSCs. Immunofluorescence detection of von Willebrand factor (red signal in Figure 24h) and Lamin A/C (green signal in Figure 24h) demonstrated that the human ADSCs delivered into a subcutaneous location of a SCID mouse participated in the formation of new blood vessels.

In the heart of the mice shown in Figure 23, we made the following observations 70 days post-delivery (all corresponding images are provided in [123]: some injected human ADSCs had differentiated into cardiomyocytes, as evidenced by co-expression of troponin I and lamin A/C (detecting human cell nuclei) in those cells (c.f. Figure 17); other injected human ADSCs had differentiated into endothelial cells, as evidenced by coexpression of endothelial cell marker vWF and lamin A/C; some injected human ADSCs maintained proliferation potential, as evidenced by co-expression of proliferating cell marker Ki67 and lamin A/C; and some injected human ADSCs underwent apoptosis, as evidenced by the presence of TUNEL-positive signals in lamin A/C positive human ADSCs [123].

Moreover, no lamin A/C signal was observed in sections of the lung, liver, kidney, spleen, and brain of these mice, indicating that intramyocardially delivered human ADSCs did, in principle, not migrate into other tissues or organs [123].

### 5.4. Specific Therapeutic Benefits of Adipose-Derived Regenerative Cells

One of the most striking features of ADRCs in cell-based therapy is their differentiation potential without any prior manipulation, genetic alteration, or the need for culturing the cells. The latter facilitates to isolate ADRCs and re-apply them to the same subject at the point of care without the need for expensive equipment, complicated processing, or repeated interventions.

It is crucial to bear in mind that, in contrast to ADSCs, UA-ADRCs in principle cannot be labeled (because this would render them modified). Accordingly, it is not possible to experimentally determine whether the following benefits of ADSCs also apply to UA-ADRCs (although it is reasonable to hypothesize that this is indeed the case). Specifically, ADSCs can (i) stay locally, survive, and engraft in the new host tissue into which the cells were applied (Figure 17 and Figure 18); (ii) differentiate under guidance of the new microenvironment into cells of all three germ layers (Figure 12, Figure 13 and Figure 14); (iii) integrate into and communicate within the new host tissue by forming direct cell–cell contacts (Figure 16, Figure 17 and Figure 18); (iv) exchange genetic and epigenetic information through release of exosomes (Figure 19); (v) participate in building new vascular structures in the host tissue (Figure 24h and [51]); (vi) positively influence the new host tissue by release of cytokines (among them vascular endothelial growth factor and insulin-like growth factor 1) [124]; (vii) protect cells at risk in the new host tissue from undergoing apoptosis [124]; and (viii) induce immune modulatory and anti-inflammatory properties [79,80], whereby the inhibiting effect on apoptosis may play an important role (79).

### 5.5. Local vs. Systemic Application of UA-ADRCs

Several studies showed that local injection of ADRCs is safe [125,126,127]. 

When stem cells are injected into the circulation, they are ‘searching’ for a place where they could be of benefit. As a tumor is considered a ‘wound that does not heal’ [128,129], it releases cytokines and other factors that aim to attract circulating stem cells to the tumor site [54,77,130], where stem cells may assist the tumor to build its stroma and thereby help the tumor to grow faster [131]. Hence, UA-ADRCs preferably should be applied locally to the side of need. In case of systemic application, the potential of UA-ADRCs to support an already existing tumor in its growth (in contrast to the absent ability of UA-ADRCs to induce a de novo tumor) should be considered, pointing to the need for evaluation of the oncogenic status of the patient prior to a systemic application. 

## 6. Examples of Application of Unmodified, Autologous, Adipose-Derived Regenerative Cells (UA-ADRCs) in Regenerative Cell Therapy

### 6.1. Tendon Defects

Our group recently published a prospective, randomized, controlled first-in-human pilot study suggesting that the use of UA-ADRCs in subjects with symptomatic, partial-thickness rotator cuff tear (sPTRCT) is safe and leads to improved shoulder function without adverse effects [127]. Specifically, we demonstrated that the risks connected with treatment of sPTRCT with UA-ADRCs were not greater than those connected with treatment of sPTRCT with corticosteroid injection. On the other hand, the subjects who were treated with UA-ADRCs showed a statistically and significantly higher mean American Shoulder and Elbow Surgeons Standardized Shoulder Assessment Form (ASES) total scores [132] at 24 weeks and 52 weeks post-treatment than those subjects who were treated with corticosteroid [127]. Based on the encouraging results of this pilot study, a respective pivotal, randomized controlled trial on 246 patients suffering from sPTRCT is currently ongoing [133].

**Figure 25:**
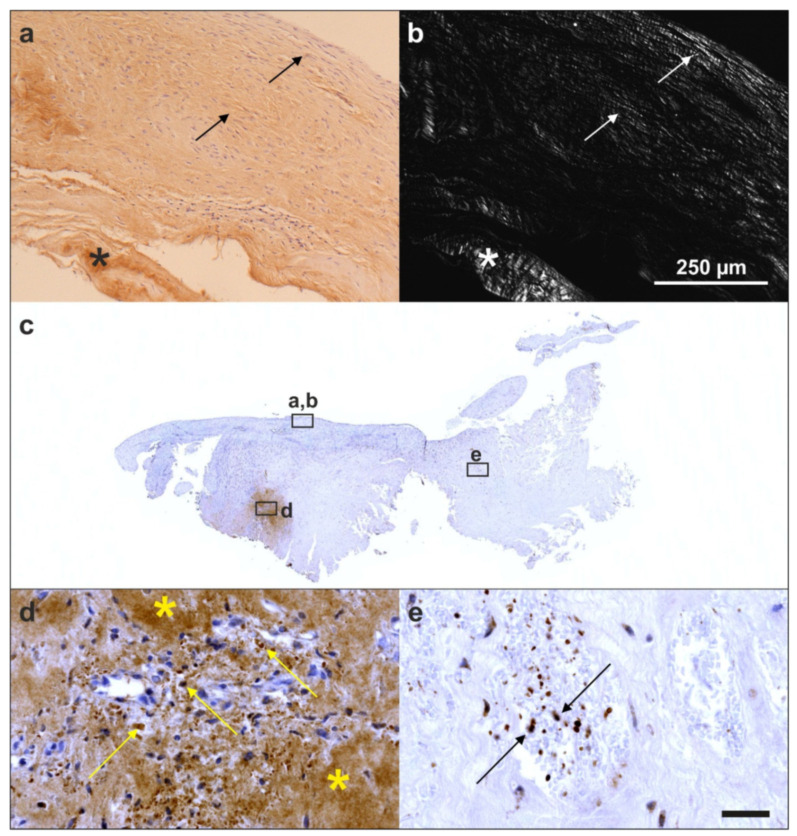
(**a**,**b**) Original (asterisk) and newly formed (arrows) tendon tissue in a section of a biopsy from a human supraspinatus tendon that was taken ten weeks post-treatment of a traumatic sPTRCT using UA-ADRCs (reprinted from [31] with permission from the sportärztezeitung) (**a**): immunohistochemical detection of type I collagen; (**b**): polarization microscopic image of the section shown in (**a**). Note the organized, slightly undulating type I collagen and the high cell density in the newly formed tendon tissue which does not resemble scar tissue (details in [134]). (**c**) Immunohistochemical detection of tenomodulin in a section of the same biopsy (section adjacent to the one shown in a) (Panels (**c**–**e**) reprinted from [134] with permission from Baishideng Publishing Group). The insets show the position of Panels (**d**) and (**e**) (as well as the position of Panels (**a**,**b**) in the adjacent section). (**d**) Position of a spot with very high density of cells and microvessels (yellow arrows, intracellular immunolabeling for tenomodulin; yellow asterisks, extracellular immunolabeling for tenomodulin). (**e**) Position of degenerative tendon tissue with formation of microvessels (black arrows, intracellular immunolabeling for tenomodulin in cells inside microvessels). The scale bar represents 100 µm in (**a**,**b**), 630 µm in (**c**), and 50 µm in (**d**,**e**).

Furthermore, we investigated a biopsy of a human supraspinatus tendon that was taken ten weeks post-treatment of a traumatic sPTRCT using UA-ADRCs [134]. Most intriguingly, the microscopic images of the tendon treated with UA-ADRCs clearly demonstrated that a different type of healing had taken place. Specifically, the formation of new tendon tissue and the absence of scar tissue (Figure 25a,b) that was observed are regenerative processes that are typically only observed in fetal tendons [134]. We also found abundant intracellular and extracellular presence of tenomodulin in a region that most probably represented the site of injection of UA-ADRCs (Figure 25c,d) (tenomodulin is a tendon-specific marker important for tendon maturation, with key implications for residing tendon stem/progenitor cells and the regulation of endothelial cell migration [135,136,137]). This finding was in line with the hypothesis that tendon regeneration observed in the investigated biopsy was ‘orchestrated’ from this region. Furthermore, the presence of tenomoduline immunopositive cells inside microvessels in another region of the biopsy (that mostly showed degenerative tendon tissue, characterized by unorganized collagen and few, rounded cells) (Figure 25e) may have indicated an unsuccessful attempt of the body to endogenously initiate tendon regeneration by transferring corresponding cells via the blood stream into the injured/degenerative tissue. 

Collectively, these data strongly support the usefulness of transferring UA-ADRCs, which have been taken from one part of the body where they are not ‘missed’ to tissue in need of regeneration, in order to re-establish tissue homeostasis (c.f. Figure 22c).

### 6.2. Cartilage Defects

**Figure 26:**
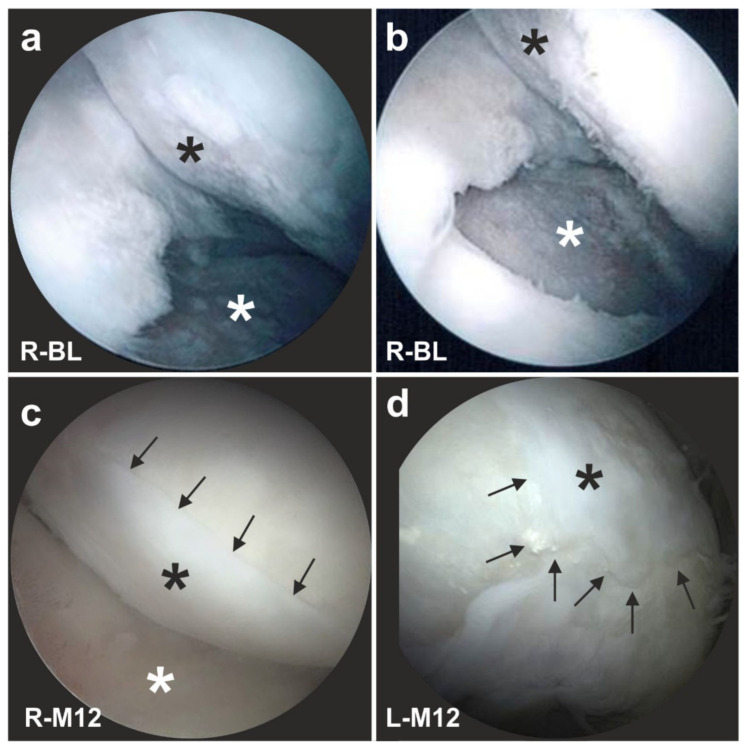
Example of successful application of unmodified, autologous, adipose-derived regenerative cells (UA-ADRCs) for treating cartilage defects (reprinted from [55] with permission from MDPI). The panels show arthroscopic views of the right (**a**–**c**) and the left (**d**) knee of a male, 51-year-old subject who presented with recurring and increasing pain in both knee joints during walking and other activities. (**a**) Third-degree damage to the right tibial plateau (white asterisk) after a tibial chondrocyte transplantation that had been performed three years previously, as well as considerable osteoarthritic damage of the femoral cartilage (black asterisk). (**b**) Situation of the right knee after arthroscopic removal of the failed chondrocyte transplant (white asterisk) as well as ‘mushy’ and damaged cartilage structure on the femoral condyles before it was removed (black asterisk). (**c**) Situation of the right knee one year after arthroscopic removal of damaged cartilage and a single application of UA-ADRCs isolated from 100 mL of lipoaspirate, showing complete healing of the tibial defect (white asterisk) and of the femoral parts, with formation of new whitish cartilage that shows a sharp demarcation border to the existing old and more yellowish cartilage (arrows). (**d**) Situation of the left knee one year after performing a standard procedure without application of UA-ADRCs (i.e., arthroscopic removal of damaged cartilage and drilling of small holes into the bone), showing a somewhat uneven, overshooting fibroblastic scar formation (black asterisk) without a sharp demarcation border to the original cartilage (arrows). Abbreviations: R, right; L, left; BL, baseline; M12, twelve months after baseline.

The advantages of treating cartilage defects with ADRCs were documented in 27 clinical trials so far, with a total number of >700 subjects treated with ADRCs (Schmitz et al.; systematic review and meta-analysis in preparation). These specific advantages are exemplified here by the following example of a male, 51-year-old subject who presented with recurring and increasing pain in both knee joints during walking and other activities (all treatments and procedures described in this section were performed in the framework of a clinical study that was approved by the Freiburg Ethics Commission International (feki; Freiburg, Germany) (feki code 013/1371)). The subject’s history included a tibial chondrocyte transplant that had been performed three years previously. Figure 26a shows an arthroscopic view of third-degree damage to the right tibial plateau where the transplanted chondrocytes were gone and only the artificial matrix with small holes implanted on the tibial plateau was still present (white asterisk in Figure 26a). Furthermore, considerable osteoarthritic damage of the femoral cartilage was observed (black asterisk in Figure 26a). Figure 26b shows the situation after arthroscopic removal of the failed chondrocyte transplant (white asterisk in Figure 26b) as well as ‘mushy’ and damaged cartilage structure on the femoral condyles before it was removed (black asterisk in Figure 26b). Then, the right knee was treated with a single application of UA-ADRCs obtained from 100 mL of lipoaspirate. 

A control arthroscopy one year later showed complete healing of the tibial defect (white asterisk in Figure 26c) and of the femoral parts, with formation of new whitish cartilage that showed a sharp demarcation border to the original, more yellowish cartilage (arrows in Figure 26c). 

The left knee of the same subject was treated with a standard procedure without application of UA-ADRCs, i.e., arthroscopic removal of damaged cartilage and drilling of small holes into the bone. A control arthroscopy one year later showed a somewhat uneven, overshooting fibroblastic scar formation (asterisk in Figure 26d) without a sharp demarcation border to the original cartilage (arrows in Figure 26d). This indicated that there was some sort of healing, but not a regrowth of organized cartilage, as we have hypothesized for the right knee after application of UA-ADRCs. 

**Figure 27:**
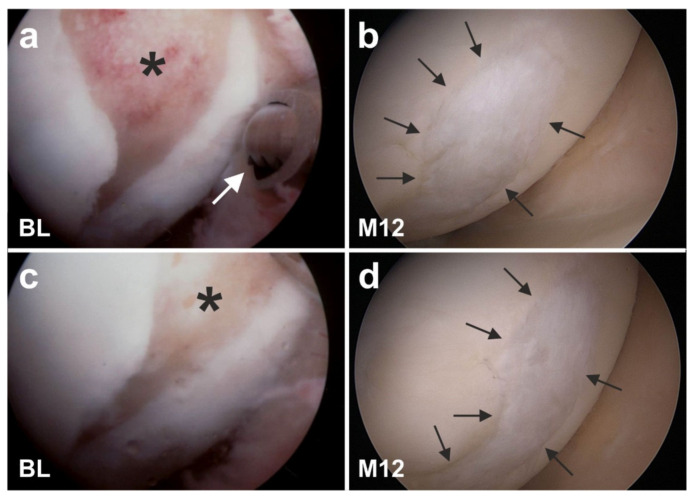
Other examples of successful application of unmodified, autologous, adipose-derived regenerative cells (UA-ADRCs) for treating cartilage defects (previously unpublished figure). The panels show arthroscopic views of the knees of two subjects (Subject 1 (male, 45 years old): (**a**,**b**); Subject 2 (male, 55 years old): (**c**,**d**) before (**a**,**c**) and one year after (**b**,**d**) arthroscopic removal of damaged cartilage (asterisks in (**a**,**c**)) and application of UA-ADRCs isolated from 50 mL of lipoaspirate. The arrows in (**b**,**d**) indicate the sharp demarcation border between the newly formed and the original cartilage. The white arrow in (**a**) points to the arthroscopic instrument that was used to remove damaged cartilage.

Figure 27 shows arthroscopic views of cartilage defects of the knees of two other subjects that were also successfully treated with UA-ADRCs. Of note, the finding of a sharp demarcation border between the newly formed cartilage and the original cartilage (arrows in Figure 26c) was found as well in these subjects one year after treatment with UA-ADRCs (arrows in Figure 27b,d). Again, we have hypothesized that this arthroscopic finding indicated regrowth of organized cartilage. 

**Figure 28:**
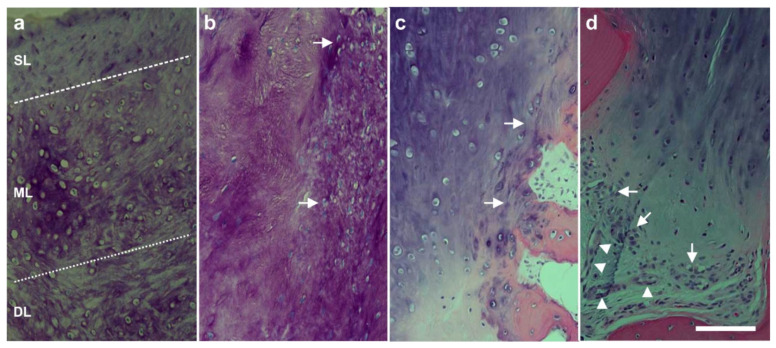
Representative photomicrographs of 5-µm-thick histologic sections (stained with toluidin blue (**a**,**b**) or hematoxylin and eosin stain (**c**,**d**)) of the small tissue samples taken during arthroscopic inspection of the knees of the subject represented in Figure 26 at one year after arthroscopic removal of damaged cartilage and a single application of unmodified, autologous, adipose-derived regenerative cells (UA-ADRCs) (right knee) (**a**,**c**) or after performing a standard procedure without application of UA-ADRCs (i.e., arthroscopic removal of damaged cartilage and drilling of small holes into the bone) (left knee) (**b**,**d**), respectively (reprinted from [55] with permission from MDPI). The dotted lines in (**a**) (knee treated with UA-ADRCs) indicate a zonal organization of the newly formed cartilage with differently shaped chondrocytes in a superficial (SL), middle (ML) and deep layer (DL). The arrows in (**b**) (knee treated with a standard procedure without application of UA-ADRCs) point to scattered cells within newly formed amorphous fibrocartilage. The arrows in (**c**) (knee treated with UA-ADRCs) indicate typical chondrocytes with a small nucleus and a hollow space around in the directly built contact zone between the newly formed cartilage and bone. In contrast, the arrows in (**d**) (treatment without UA-ADRCs) point to an infiltration with inflammatory cells in the contact zone between a newly formed fibro-cartilage and bone. The arrowheads in (**d**) indicate three small blood vessels. The scale bar represents 100 µm in (**a**–**d**).

To test the hypothesis that the sharp demarcation borders between the newly formed and the original cartilage (arrows in Figure 26c and Figure 27b,d) indicated regrowth of organized cartilage after application of UA-ADRCs, we obtained written, informed consent by the subject represented in Figure 26 which stated that small tissue samples could be taken from the regenerated tissue during the follow-up arthroscopy. Histologic analysis of the tissue samples showed two key differences between the samples. (i) After application of UA-ADRCs, the newly formed cartilage showed (such as in a textbook of histology [138]) a zonal organization with differently shaped chondrocytes in a superficial, middle, and deep layer (Figure 28). In contrast, without application of UA-ADRCs, a more amorphous fibrocartilage with scattered cells (arrows in Figure 28b) was achieved that had no such layered organization (Figure 28b). (ii) Furthermore, after application of UA-ADRCs, the contact zone between the newly formed cartilage and bone showed (again, such as in a textbook of histology) typical chondrocytes with a small nucleus and a hollow space around (arrows in Figure 28c). In contrast, without application of UA-ADRCs, the contact zone between the newly formed cartilage and bone showed an infiltration with inflammatory cells, fibroblasts (arrows in Figure 28d) and small blood vessels (arrowheads in Figure 28d).

**Figure 29:**
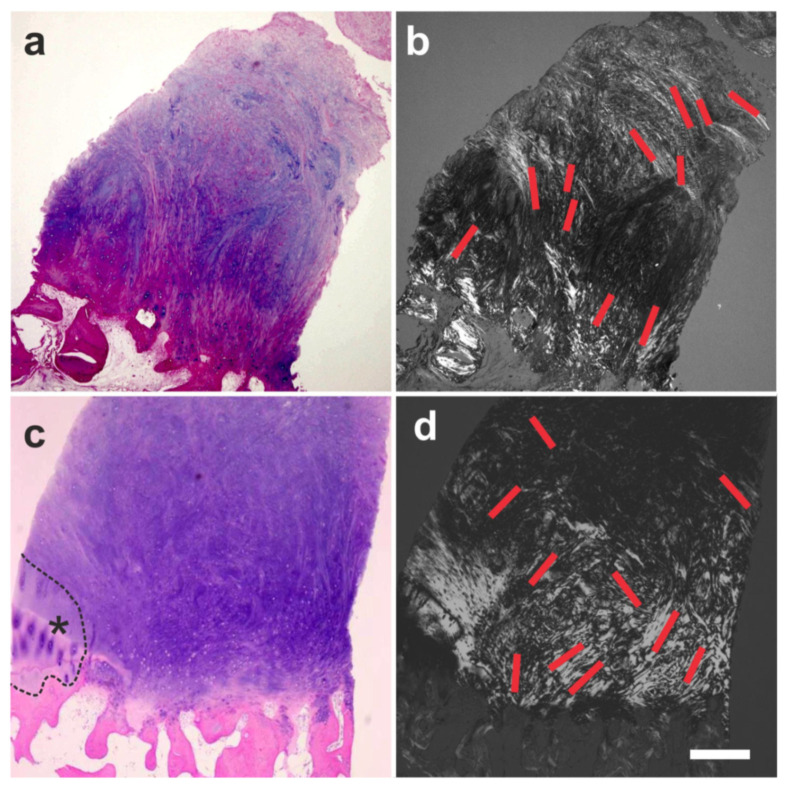
Representative photomicrographs of 5-µm-thick histologic sections (stained with hematoxylin and eosin stain) of the small tissue sample taken during arthroscopic inspection of the right knee of the subject represented in Figure 26 at one year after arthroscopic removal of damaged cartilage and a single application of the subject’s own, unmodified, autologous, adipose-derived, regenerative cells (previously unpublished figure). The panels (**b**,**d**) were generated with polarized light microscopy and converted to grayscale. The red lines in (**b**,**d**) indicate the orientation of the collagen fiber bundles within the newly formed cartilage (more vertically in in the deep and middle layers, and more horizontally in the superficial layer). The asterisk in (**c**) indicates a region of contact between the original cartilage and bone, demonstrating that the tissue sample was indeed taken from the newly formed cartilage, including the original border zone between bone at the bottom and newly formed cartilage on top (dotted line). The scale bar represents 100 µm in (**a**–**d**).

Analysis of the tissue sample taken during arthroscopic inspection of the right knee of the subject represented in Figure 26 at one year after arthroscopic removal of damaged cartilage and a single application of UA-ADRCs with polarized light microscopy demonstrated that the collagen fiber bundles in the deep and middle layers of the newly formed cartilage showed a more vertical orientation (perpendicular to the border between bone and cartilage), whereas the collagen fiber bundles in the superficial layer showed a more horizontal orientation (parallel to the surface) (Figure 29). This finding is in line with the description of the physiologic orientation of collagen fiber bundles in articular cartilage in the literature when analyzed with polarized light microscopy [139]. 

To our knowledge, the results presented in this section go beyond the state-of-the-art in the field of regenerating damaged cartilage with ADRCs and ADSCs. In a recent review [140], a number of clinical studies were listed in which cartilage defects in the human knee were treated with ADSCs [141,142,143]. Of note, in all of these studies, *cultured* ADSCs were applied, whereas we have been using *fresh, uncultured* ADRCs (for the differences and advantages of ADRCs over ADSCs see Section 5.4). The maximum follow-up period in [141,142] was only six months after application of ADSCs (MRI, arthroscopy, and histologic analysis in both studies; n = 18 subjects in both studies). In the single case report [143], MRI was performed at twelve months after application of ADSCs, but no arthroscopy and, thus, no histologic analysis were performed. Furthermore, in none of these studies tissue samples were investigated using polarized light microscopy.

### 6.3. Chronic, Recalcitrant Low Back Pain Caused by Lumbosacral Facet Syndrome

**Figure 30:**
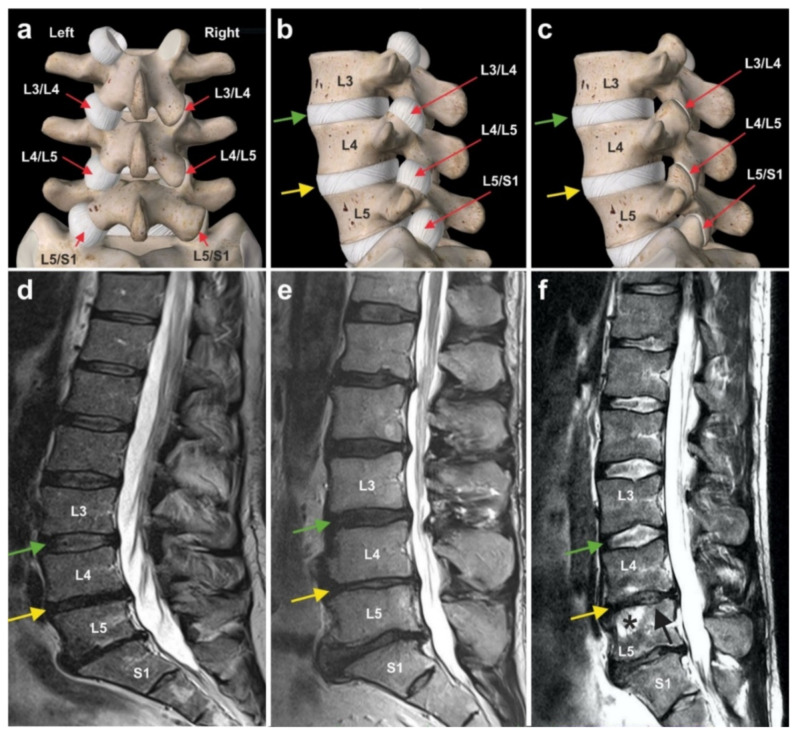
(**a**–**c**) Schematic of the lumbar vertebrae L3 to L5 and the upper edge of the os sacrum of a human spine from dorsal (**a**) and lateral (**b**,**c**) (previously unpublished figure). The red arrows indicate the zygapophyseal (facet) joints between L3 and L4 (L3/L4), between L4 and L5 (L4/L5) and between L5 and the os sacrum (L5/S1) with joint capsule (on the left side in (**a**,**b**)) as well as without joint capsule (on the right side in (**a**,**c**)). The green arrows in (**b**,**c**) indicate the intervertebral disc between L3 and L4, and the yellow arrows the intervertebral disc between L4 and L5. (**d**–**f**) MRI scans of the lumbar spine (from lateral) of three former professional, internationally highly successful ski racers (we are not allowed to provide more information about these subjects in order to protect privacy). The green arrows indicate normal intervertebral disc structure between L3 and L4, whereas the yellow arrows show that, in all three athletes, there was a reduction in the height of the intervertebral disc (and, thus, the intervertebral space) between L4 and L5. The asterisk in (**f**) points to a bone marrow edema in the vertebral body of L5, and the black arrow in (**f**) to an upper plate collapse of L5. All three athletes were treated with unmodified autologous adipose-derived regenerative cells. The cells were injected at the site of the right and left facet joints between L4 and L5 as well as between L5 and S1 within a single procedure of harvesting and injection that lasted about two hours. This treatment resulted in significant and long-lasting (now more than three years) pain reduction that enabled one of these athletes to very successfully return to competitive sports.

Lumbosacral facet syndrome is a term used to describe a painful condition caused by inflammation and irritation of the zygapophyseal (facet) joints of the spine [144] (Figure 30a–c). It is most commonly caused by degenerative changes in the lumbosacral spine, and predominantly the consequence of a reduction in height of a lumbar disc, following a previous disc prolaps. As a consequence, the distribution of body weight (that normally rests on the vertebral column and the discs) is now also resting on the facet joints that are made for elasticity but not for weight load bearing. As a consequence of this overload, the small facet joints become chronically inflamed. Symptoms include low back pain with or without referral to the lower extremities [144]. To our knowledge, reports on cell-based therapies for chronic low back pain caused by lumbosacral facet syndrome have not yet been published.

Figure 30d–f shows typical wear and tear that occurred in the spine of three former professional ski racers (all treatments and procedures described in this section were performed in the framework of clinical assessment). The green arrows indicate normal intervertebral disc structure between the lumbar vertebrae L3 and L4, demonstrating that there was a certain level of water content, reflected by the white signal in the MRI. In contrast, the yellow arrows show that between vertebrae L4 and L5 in all three athletes there was a reduction in height of the intervertebral disc due to the diminished overall volume of the intervertebral disc. The increased body weight now resting on the facet joints resulted in an inflammatory reaction of the facet joints. All three athletes were treated with UA-ADRCs. The cells were injected at the site of the right and left facet joints between L4 and L5 as well as between L5 and S1 within a single procedure that lasted about two hours from harvesting the adipose tissue to injecting the cells. This treatment resulted in significant and long-lasting (now more than three years) pain reduction that enabled one of these athletes to very successfully return to competitive sports.

**Figure 31:**
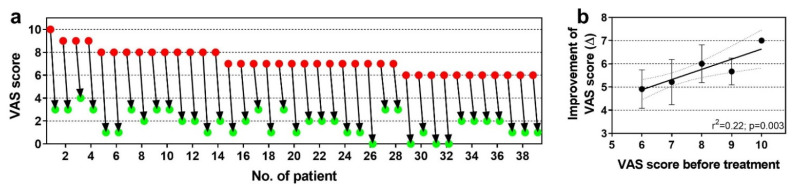
(**a**) Connected individual VAS scores of n = 39 subjects with chronic, recalcitrant low back pain caused by lumbosacral facet syndrome before (red dots) and after (green dots) treatment with UA-ADRCs isolated from 100 mL of lipoaspirate each (the follow-up data ranged between one and more than three years) (previously unpublished figure). The subjects were listed consecutively according to the date of treatment. (**b**) Mean and standard deviation of the individual improvement of the VAS score of the subjects shown in (**a**) as a function of the VAS score at baseline (i.e., before treatment). The results of linear regression analysis (regression line with 95% confidence interval) are also indicated (r^2^ = 0.22; *p* = 0.003).

Figure 31a shows individual pain scores on a VAS scale (with 0 representing no pain and 10 representing maximum, unbearable pain) of n = 39 subjects with chronic, recalcitrant low back pain caused by lumbosacral facet syndrome before (red dots in Figure 31a) and one year after (green dots in Figure 31a) treatment with UA-ADRCs isolated from 100 mL of lipoaspirate each (the follow-up interval ranged between one and more than three years) (modified from [125] (all treatments and procedures described in this section were performed in the framework of a clinical study that was approved by the Freiburg Ethics Commission International (feki; Freiburg, Germany) (feki code 016/1252)). The mean and standard error of the mean of the VAS scores before (red dots) and after (green dots) treatment was 7.21 ± 0.17 and 1.80 ± 0.17; this difference was highly statistically significant (Wilcoxon matched-pairs signed rank test; *p* < 0.001). As a consequence, the quality of life of these subjects was substantially improved. There was no single subject that was not impressively benefitting from the treatment.

Figure 31b shows the mean and standard deviation of the individual improvement of the VAS score after treatment as a function of the VAS score before treatment of these 39 subjects. Linear regression analysis showed a statistically significant relationship between the VAS score before treatment and the individual improvement of the VAS score after treatment, with the best results obtained with the highest VAS scores before treatment (r^2^ = 0.22; *p* = 0.003). This impressively demonstrates the potential of UA-ADRCs for the treatment of chronic, recalcitrant low back pain caused by lumbosacral facet syndrome, and perhaps opens up the possibility for obtaining comparable results for other chronic pain conditions of the musculoskeletal system. A corresponding randomized controlled trial is currently ongoing [145].

### 6.4. Guided Bone Regeneration (Individual Case Reports)

**Figure 32:**
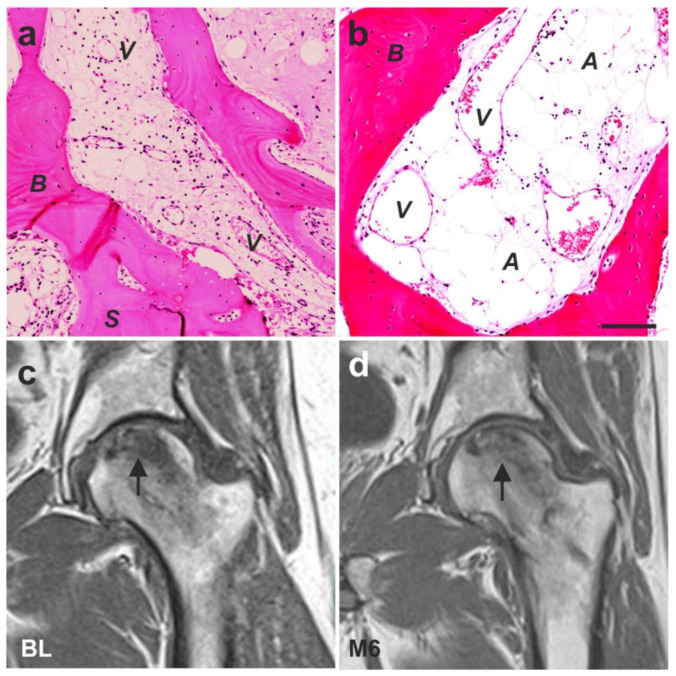
(**a**,**b**) Representative photomicrographs of 3-µm-thick paraffin sections stained with hematoxylin and eosin stain of biopsies that were collected at 34 weeks after performing guided bone regeneration (in the framework of a bilateral external sinus lift procedure as well as a bilateral lateral alveolar ridge augmentation in a 79-year-old subject who presented with a partly failing maxillary dentition) using a combination of approximately 50 × 10^6^ unmodified, autologous, adipose-derived regenerative cells (UA-ADRCs), Fraction 2 of plasma rich in growth factors (PRGF-2) and an osteoinductive scaffold (OIS) (right side) (**a**) or a combination of PRGF-2 and the same OIS alone (left side) (**b**) (reprinted from [146] with permission from Baishideng Publishing Group). Abbreviations: *A*, adipocytes; *B*, newly formed bone; *S*, scaffold; *V*, veins. The scale bar represents 100 µm in (**a**,**b**). (**c**,**d**) MRI scans of the left hip of a 41-year-old male subject suffering from avascular necrosis of the femoral head (arrows in (**c**,**d**)) at baseline (**c**) and at six months after two applications of UA-ADRCs (previously unpublished images).

The promising results achieved in the treatment of cartilage defects and chronic, recalcitrant low back pain caused by lumbosacral facet syndrome (described in detail in Section 6.2 and Section 6.3) gave reason to hypothesize that the application of UA-ADRCs could also advance the treatment of other pathologies of the musculoskeletal system. In this regard, it was a key finding that, in guided bone regeneration (GBR) (exemplified by a case of a 79-year-old subject who presented with a partly failing maxillary dentition and who was treated with a bilateral external sinus lift procedure as well as a bilateral lateral alveolar ridge augmentation), the combined application of UA-ADRCs, Fraction 2 of plasma rich in growth factors (PRGF-2), and an osteoinductive scaffold (OIS) (Treatment A), was superior to the combination of PRGF-2 and the same OIS alone (Treatment B) [146] (these treatments and procedures were performed in the framework of a clinical study that was approved by the Ethics Committee of the Federal Dental Association Hamburg (Hamburg, Germany) (no. PV5211)). Specifically, Treatment A resulted in faster buildup of higher relative amounts (area/area) of newly formed bone, connective tissue and arteries as well as in lower relative amounts of adipocytes and veins at 34 weeks after GBR (Figure 32a,b) compared to standard treatment without stem cells.

Avascular necrosis of the femoral head is one of the many indications where bone regeneration is essential for rehabilitation [147]. Cell-based therapy for this pathology has been addressed in a number of clinical studies (for a recent systematic review and meta-analysis see [148]). One of these studies was a case report of a 43-year-old male subject who was successfully treated with ADRCs mixed with platelet-rich plasma and hyaluronic acid [149]. We went one step further and treated a 41-year-old male subject suffering from avascular necrosis of the femoral head only with ADRCs (all treatments and procedures were performed in the framework of clinical assessment). Figure 32c shows an MRI scan of the left hip of this subject who was confined to a wheelchair because of unbearable pain during walking, with a significant necrotic space in the head of the subject’s left femur. Six months after two applications of UA-ADRCs (that were locally injected through a channel which came through the lateral side of the greater trochanter), a control MRI scan showed a markedly improved situation (Figure 32d), and the subject could leave the wheelchair and walks now without pain.

### 6.5. Examples of Other Treatments with UA-ADRCs (Individual Case Reports)

**Figure 33:**
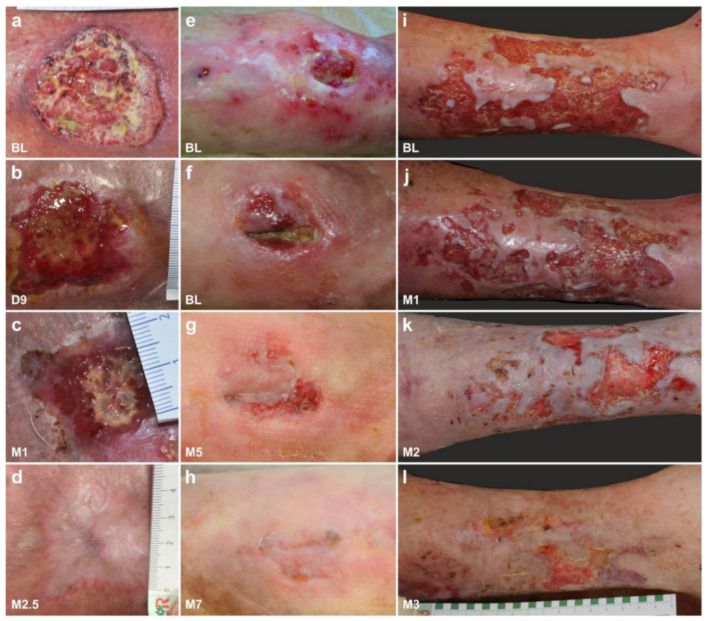
Examples of successful application of unmodified autologous adipose-derived regenerative cells (UA-ADRCs) for treating chronic wounds that did not heal for years (previously unpublished figure). (**a**–**d**) Female, 76-year-old subject; chronic wound over the medial malleolus; single application of UA-ADRCs isolated from 30 mL of lipoaspirate. (**e**–**h**) Male, 82-year-old subject; chronic wound on the lower leg caused by a phosphorus bomb during World War II; single application of UA-ADRCs isolated from 30 mL of lipoaspirate. (**i**–**l**) Female, 84-year-old subject, chronic wound on the lower leg; single application of UA-ADRCs isolated from 30 mL of lipoaspirate. Abbreviations: BL, baseline; D9, nine days after application of UA-ADRCs; M1/M2/M2.5/M3/M5/M7, one/two/two and a half/ three/five/seven months after application of UA-ADRCs.

Several studies on animal models and clinical pilot studies have shown that human ADRCs and ADSCs are able to enhance and accelerate wound healing, especially in chronic wounds [126,150,151]. An example of successful application of UA-ADRCs for treating chronic wounds in humans is shown in Figure 33, which represent cases of prolonged wounds that did not heal for several years (these treatments and procedures were performed in the framework of a clinical study that was approved by the Ethics Committee of the Medical Faculty of the Technical University Munich (Munich, Germany) (no. 5639/12)). The underlying principle in those wounds is that the regenerative power of the local stem cells is exhausted due to the repeated futile attempts at healing. Debridement of the wounds showed that debrided tissue contained only very few living cells. The transfer of UA-ADRCs activated the wounds after a few days and induced shrinkage and significant reduction in the inflammatory redness. Typically, after two to three months, all wounds based on venous insufficiency were closed. Of note, the images shown in Figure 33 impressively demonstrate that UA-ADRCs do not only work in younger people. In one 85-year-old subject who suffered from open leg wounds of more than 100 cm^2^ for several years, healing was obtained after a single application of UA-ADRCs (Figure 33c).

**Figure 34:**
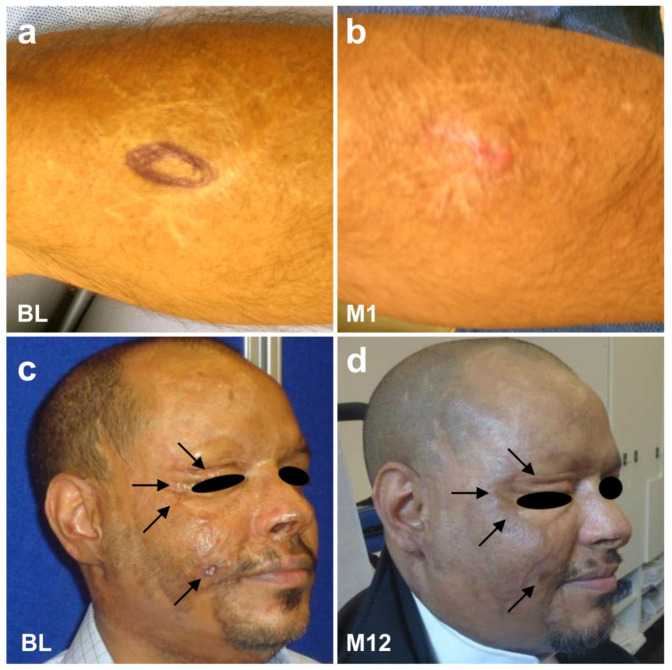
Examples of successful application of unmodified, autologous, adipose-derived regenerative cells (UA-ADRCs) for reducing scar tissue (previously unpublished figure). (**a**,**b**) Male, 48-year-old subject; scar tissue formation on the upper arm after an injury caused by a car accident; single application of UA-ADRCs isolated from 100 mL of lipoaspirate. (**c**,**d**) Male, 50-year-old subject; scar tissue formation (arrows) in the face after an acid attack; three treatments with UA-ADRCs isolated from 100 mL of lipoaspirate on days 1, 90, and 180. Abbreviations: BL, baseline; M1/M12, one/twelve months after application of UA-ADRCs.

Primarily not considered a mainstream indication for stem cell therapy, there is anecdotal evidence indicating that UA-ADRCs have a great effect on remodeling of scar tissue. As demonstrated in Figure 34, the degradation of the extracellular matrix, which leads to scarring, can be reversed (all treatments and procedures described in this section were performed in the framework of clinical assessment). We still do not fully understand the exact mechanisms behind this effect [151,152]. However, the expression of MMP2 may enable ADRCs to migrate through tissue [134], and subsequent expression of MMP9 might be responsible for the realignment of the extracellular matrix [134,152].

**Figure 35:**
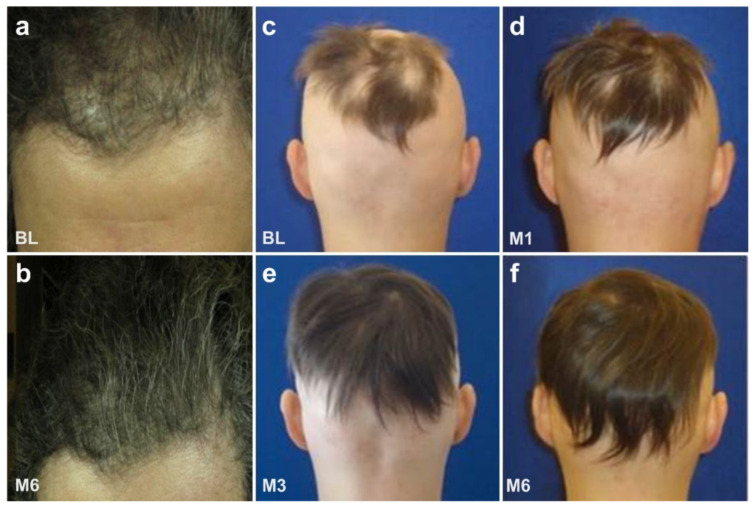
Examples of successful application of unmodified, autologous, adipose-derived regenerative cells (UA-ADRCs) for treating hair loss (previously unpublished figure). (**a**,**b**) 46-year-old female subject; sparse hair; single application of UA-ADRCs isolated from 100 mL of lipoaspirate. (**c**–**f**) 23-year-old female subject suffering from atopic dermatitis and alopecia totalis for three years without spontaneous improvement; three treatments with UA-ADRCs isolated from 100 mL of lipoaspirate each at baseline (**c**) and at three and six month after the first treatment. Results are shown at one (M1; (**d**)), three (M3; (**e**)) and seven (M7; (**f**)) month after the first treatment.

Figure 35 shows the effects of UA-ADRCs on hair growth (all treatments and procedures described in this section were performed in the framework of clinical assessment). The mechanism is assumed to be similar to the regeneration of an organ or other tissues. Specifically, we hypothesize that the delivered stem cells most likely ‘were guided’ by the remaining hair roots to their ‘local task’ of trans-differentiation into hair. 

## 7. Conclusions

Regenerative medicine and cell therapy are not yet part of mainstream clinical practice. Therapies based on UA-ADRCs discussed in this paper appear to be highly promising candidates for repair and regeneration of many tissues and ultimately for wide adoption to the practice of medicine. One of the most striking features of UA-ADRCs is their differentiation potential without any prior modification or need for culturing. Furthermore, UA-ADRCs can be obtained from a small amount of adipose tissue when using the appropriate, enzyme-supported technology for isolating vaPS cells. The fact that tissue can be harvested from and cells can be re-applied to the same subject at the point of care in one clinical session without the need for expensive equipment, complicated processing, or repeated interventions indicates easy integration into the clinical workflow.

As with any medical innovation, the scientific and medical community interested in these novel therapies needs to develop sound clinical evidence to further show safety and efficacy of cell-based therapies. Our understanding of the mechanism of actions and potential benefit of stem cell therapy has increased enormously over the past decade and we hope that there is now enough data to convince others to embark on scientifically designed clinical studies that will provide the necessary objective evidence. Especially musculoskeletal indications with their large incidence and prevalence rates and often substantial total cost of care associated with current clinical practice should prove to be attractive candidates for such efforts.

An important factor for successful implementation of therapies using UA-ADRCs will be the proactive support of regulatory authorities to design frameworks that, while addressing valid concerns around the safety of unproven therapies that can be found in some places currently [153], show a clear path to market approval and reimbursement. Some progress in that direction can be observed in a number of jurisdictions. For example, the US FDA has provided clear guidance for Regenerative Medicine Advanced Therapies in 2017. Other countries, such as Japan and Australia, also seem to have created an advanced regulatory framework that will allow subjects to gain fast access to regenerative medicine. Unfortunately, the situation in the European Union (EU) is not yet that clear. A complex set of guidance and regulations that result in somewhat convoluted responsibilities between the EU, member states, and local authorities will need to eventually be streamlined to help to make these promising therapies available for all subjects in the EU too. 

It is our hope that this paper will help to clarify and facilitate the understanding, development, and adoption of regenerative medicine in general and specifically of therapies based on UA-ADRCs. Not all medical challenges that we face in clinical practice today will be candidates for regenerative medicine. However, as we are facing more and more age-related diseases, we are convinced that therapies using UA-ADRCs will play a fundamental role in a continuously increasing number of indications. This has the potential to trigger the development of an entire new generation of medicine for the benefit of subjects and of healthcare systems.

## Data Availability

There are no additional data available that support the results shown in Figure 1, Figure 2, Figure 3, Figure 4, Figure 5, Figure 6, Figure 7, Figure 8, Figure 9, Figure 10, Figure 11, Figure 12, Figure 13, Figure 14, Figure 15, Figure 16, Figure 17, Figure 18, Figure 19, Figure 20, Figure 21, Figure 22, Figure 23 and Figure 24 except for the corresponding references provided in the figure legends. The patient whose results are shown in Figure 25 was the first author of this paper (E.U.A.) and can be contacted for more information. On the other hand, due to confidentiality reasons, there is no data related to the results shown in Figure 26, Figure 27, Figure 28, Figure 29, Figure 30, Figure 31, Figure 32, Figure 33, Figure 34 and Figure 35 that can be shared.
